# Whole-genome sequencing-based prediction and analysis of antimicrobial resistance in *Yersinia enterocolitica* from Ningxia, China

**DOI:** 10.3389/fmicb.2022.936425

**Published:** 2022-07-22

**Authors:** Yuan Yue, Mei Shen, Xiang Liu, Qiong Hao, Yutong Kang, Yanlin Che, Fang Li, Shenglin Chen, Shuai Xu, Huaiqi Jing, Zhen-jun Li, Xue-zhang Zhou

**Affiliations:** ^1^Key Laboratory of the Ministry of Education for the Conservation and Utilization of Special Biological Resources of Western China, Ningxia University, Yinchuan, China; ^2^State Key Laboratory for Infectious Disease Prevention and Control, National Institute for Communicable Disease Control and Prevention, Chinese Center for Disease Control and Prevention, Beijing, China; ^3^Ningxia Hui Autonomous Region Food Testing and Research Institute, Yinchuan, China; ^4^Ningxia Hui Autonomous Region Centre for Disease Control and Prevention, Yinchuan, China

**Keywords:** *Yersinia enterocolitica*, whole-genome sequencing, multidrug-resistant, plasmid, antimicrobial resistance

## Abstract

Focusing on resistance trends and transmission patterns of pathogenic microorganisms is a major priority for national surveillance programs. The use of whole-genome sequencing for antimicrobial susceptibility testing (WGS-AST) is a powerful alternative to traditional microbiology laboratory methods. *Yersinia enterocolitica* antimicrobial resistance (AMR) in the Ningxia Hui Autonomous Region has yet to be described thoroughly in current studies. We assessed and monitored the development of *Y. enterocolitica* AMR in the Ningxia Hui Autonomous Region during 2007–2019 based on WGS-AST. Resistance genotypes were predicted based on WGS. Antimicrobial resistance testing using classical microbiology determined resistance to 13 antimicrobial agents in 189 *Y. enterocolitica* isolates from Ningxia. The highest resistance level was 97.88% for cefazolin, followed by ampicillin (AMP) (44.97%), ciprofloxacin (CIP) (25.40%), streptomycin (STR) (11.11%), and tetracycline (TET) (10.58%). Isolates emerged as chloramphenicol (CHL) and trimethoprim/sulfamethoxazole (SXT) resistant. The primary plasmid types were IncFII(Y) and ColRNAI. The TET, STR, and SXT resistance were mediated by the *tet*A, *aph*(6)-Id, *aph*(3″)-Ib, and *sul*2 genes located on the IncQ1 plasmid. The resistant strains were predominantly biotype 4/O:3/ST429 and the hosts were pigs and patients. The number of multidrug-resistant (MDR) strains was of concern, at 27.51%. At present, the prediction of antimicrobial resistance based on WGS requires a combination of phenotypes. From 2007 to 2019, *Y. enterocolitica* isolates from the Ningxia Hui Autonomous Region showed a relatively high rate of resistance to cefazolin (CZO) and some resistance to AMP, CIP, STR, and TET. CIP, SXT, and TET showed a relatively clear trend of increasing resistance. Plasmids carrying multiple drug resistance genes are an important mechanism for the spread of antimicrobial resistance. Isolates with low pathogenicity were more likely to present an AMR phenotype than non-pathogenic isolates.

## Introduction

Antimicrobial resistance (AMR) has been recognized as one of the principal health threats to humans and animals worldwide. The factors that cause AMR are complex and are multidimensional issues involving human and animal health, food hygiene, and environmental science (Butaye et al., 2014). There is no denying that the irrational or inappropriate use of antimicrobial agents in humans, agriculture, and veterinary medicine is strongly associated with AMR. The crucial point of this link is mobile genetic elements (MGEs) (Woolhouse et al., 2015) that lead to the transmission of AMR through the food chain or the environment, posing a serious threat to human health (Van Boeckel et al., 2015) while increasing the cost and risk of treatment.

*Yersinia enterocolitica* is widely distributed in nature and is a human-animal pathogen. It can be contacted via the fecal-oral route through contaminated food and water or contact with infected animals (Ahmed et al., 2019). *Y. enterocolitica* is heterogeneous and consists of six biotypes: 1A, 1B, 2, 3, 4, and 5. Biotype 1B was regarded as highly pathogenic, and biotype 1A was regarded as being non-pathogenic in humans; other biotypes are considered to have low pathogenicity (Valentin-Weigand et al., 2014). Yersiniosis has been reported globally, with the most severe epidemic in Europe, where it was the third most common zoonotic disease in 2019 (European Food Safety Authority, and European Centre for Disease Prevention and Control, 2021). Yersiniosis is sporadic in China, and breeding farms and slaughterhouses show high levels of contamination by *Y. enterocolitica* (Liang et al., 2012). *Y. enterocolitica* produces β-lactamases and is therefore naturally resistant to AMP, amoxicillin, and first-generation cephalosporins (Fredriksson-Ahomaa et al., 2012). The results of previous studies have shown that *Y. enterocolitica* (isolated from animal feces, raw/cooked livestock, poultry meat, and frozen food) in China has a high rate of resistance to AMP, cephalothin, SXT, amoxicillin/clavulanic acid, nalidixic acid, and CHL, as well as the presence of multidrug resistance (Sun et al., 2013; Ye et al., 2015; Peng et al., 2018). However, current studies were limited to a certain category of samples or a specific geographical area.

Traditional laboratory methods are gradually being replaced by whole-genome sequencing (WGS) because they do not provide comprehensive screening for all AMR genes, let alone identify novel AMR genes and uncertain AMR mechanisms. Currently, many studies have obtained genotypes for predicting bacterial resistance based on WGS (Zhao et al., 2015; McDermott et al., 2016; Karlsson et al., 2021). Relying on WGS allows for a more comprehensive analysis of bacterial resistance mechanisms than traditional methods alone, enabling a better correspondence between genotype and phenotype as well as the development of a molecular antimicrobial susceptibility system (Cirillo et al., 2017; Su et al., 2019). This study assessed and monitored the development of *Y. enterocolitica* AMR in the Ningxia Hui Autonomous Region during 2007–2019 based on WGS-AST. The correlation between genotype and phenotype was investigated to identify the risk of novel AMR genes and improve our understanding of the mechanisms underlying AMR.

## Materials and methods

### Bacterial isolation and identification

The 189 isolates in this experiment were obtained from five prefectures in the Ningxia Hui Autonomous Region [Yinchuan (*n* = 85), Shizuishan (*n* = 6), Wuzhong (*n* = 1), Guyuan (*n* = 3), and Zhongwei (*n* = 94)], and the study period took place from 2007 to 2019. The isolates were isolated from animal, food, and human clinical samples. Animal samples were mainly obtained from feces (*n* = 73), pharyngeal swabs (*n* = 33), anal swabs (*n* = 7), and intestinal contents (*n* = 39). The food samples (*n* = 24) were mostly derived from meat. Patient samples (*n* = 13) were obtained from patient feces. Strain enrichment was performed in phosphate-buffered saline with sorbitol and bile salts (PBS) at 4°C for 21 days, and *Yersinia* was inoculated onto CIN Agar (Cefsulodin, Irgasan, Novobiocin) (Oxoid, Basingstoke, UK/HKM, Guangzhou, China). A typical bulls-eye appearance (deep red centers surrounded by outer transparent zones) on CIN-selective agar plates was inoculated onto Kligler iron and urea media. We identified strains using the API20E biochemical identification system. The strains were further distinguished by serotyping (*Y. enterocolitica* antisera set from the Institute of Chinese Biomedicine) (Wang et al., 2009) and biotyping (Bile Aesculin Agar; Oxoid, Basingstoke, United Kingdom. Brain Heart Infusion Agar; Oxoid, Basingstoke, United Kingdom. Tween 80; Amresco, United States. CaCl_2_; Sinopharm Chemical Reagent Co., Ltd., China. Biochemical Reaction Tablets; Rosco, Denmark) (Xiao et al., 2010). The identification of biotypes is shown in Table 1. Lipase activity: freshly cultured strains were inoculated on BHI plates containing 1% Tween 80 and 0.01 % CaCl_2_. The plates were incubated for 4 days at 35°C and were positive for white turbidity around the colonies. Salicin: the strains were spotted on bile aesculin agar and incubated at 25°C for 24 h. The strains that darken the medium around the spot species were biotype 1A, the other biotypes were not colored. Other biochemical reactions were identified using biochemical tablets. Follow the protocol for fractionated tablets: prepare a suspension of fresh strains (*OD* = 3.3) in 1.5 mL Eppendorf tubes of 250 μL each and incubate the tablets overnight at 25°C. The results were determined by observing the color change.

**TABLE 1:** Biochemical tests used to biotype *Y. enterocolitica* strains (Bottone, 1997).

Test	Biogroup reaction					
	1A	1B	2	3	4	5
Lipase activity	+	+	–	–	–	–
Salicin	+	–	–	–	–	–
Esculin hydrolysis	+/–	–	–	–	–	–
Xylose	+	+	+	+	–	v
Trehalose	+	+	+	+	+	–
Indole production	+	+	v	–	–	–
Ornithine decarboxylase	+	+	+	+	+	+ (+)
Voges-Proskauer test	+	+	+	+	+	+ (+)
Pyrazinamidase activity	+	–	–	–	–	–
Sorbose	+	+	+	+	+	–
Inositol	+	+	+	+	+	+
Nitrate reduction	+	+	+	+	+	–

+, positive; −, negative; (+), delayed positive; v, variable.

### Antimicrobial susceptibility testing

Minimum inhibitory concentration (MIC) tests were performed in *Y. enterocolitica* isolates with micro dilutions in microtiter 96-well plates (AST Panel For *Aerobic Gram-Negative bacilli* E-JIN-YEA, Xingbai, Shanghai, China). The isolates were incubated on Mueller-Hinton broth at 37°C for 24 h. Fresh cultures were prepared in suspension with 0.9% NaCl and adjusted for turbidity to 0.5 McFarland’s standard.

The suspension was diluted 200-fold with Nutrient Broth Culture and blended. A 100 μL dilution was added to each well. Negative control well was added to 100 μL Nutrient Broth Culture Solution. 96-well plates were incubated at 35°C for 18–20 h.

The MIC of the *Y. enterocolitica* isolates to ampicillin (AMP), ampicillin/sulbactam (SAM), tetracycline (TET), cefazolin (CZO), cefuroxime (CXM), ceftazidime (CAZ), trimethoprim/sulfamethoxazole (SXT), polymyxin (POL), ciprofloxacin (CIP), gentamicin (GEN), streptomycin (STR), chloramphenicol (CHL), and imipenem (IPM) were determined. In clinical practice, these antibacterial agents are commonly applied in the treatment of intestinal infections. The dilutions ranged from 0.06 to 152 μg/mL. Results were only accepted if controls were functional and the duplicates did not deviate more than one dilution. Minimum inhibitory concentrations (MICs) and breakpoints were determined and interpreted according to the Clinical and Laboratory Standards Institute (CLSI) guidelines (M100-S30) (CLSI, 2020). Streptomycin was used with breakpoints as defined by the US Food and Drug Administration (FDA) (Hu et al., 2017; Supplementary Table 1). Isolates resistant to three or more antimicrobial agents were considered multi-drug resistant (MDR). The quality control strains used were *Escherichia coli* ATCC25922, *Pseudomonas aeruginosa* ATCC27853, and *Enterococcus faecalis* ATCC29212.

### Whole-genome sequencing

Genomic DNA was extracted using a Wizard^®^ Genomic DNA Purification Kit (Promega, United States) according to the manufacturer’s protocol. DNA concentration, quality, and integrity were determined using a 5400 Fragment Analyzer System (Agilent, United States) and a NanoDrop 2000 Spectrophotometer (Thermo Fisher Scientific, United States). In total, 189 isolates were sequenced using Illumina Nova technology (Supplementary Table 2). Raw reads were quality filtered and assembled. Reads containing the following parameters were removed: reads containing more than 40% low-quality bases (mass value ≤ 20); reads where the proportion of N was greater than 10%; overlap with adapter sequence more than 15 bp and less than three mismatches between the two. Assemblies were performed using SOAP denovo^1^ (Li et al., 2009), SPAdes,^2^ and Abyss^3^ software. Assembly results were integrated using CISA software^4^. Preliminary assembly results were supplemented with gapclose software to remove homologous contamination with readings of less than 0.35 of the mean depth and obtain the final assembly results.

### Sequence types and core genome multilocus sequence types determination

Sequence types (STs) and the core genome multilocus sequence types (cgMLST, CTs) of *Y. enterocolitica* isolates were assigned using EnteroBase^5^ (Zhou et al., 2020). The scheme used for STs was the McNally 7 Gene, including *aar*F, *dfp*, *gal*R, *gln*S, *hem*A, *rfa*E, and *spe*A (Hall et al., 2015). The generation and annotation of a Minimum Spanning Tree (MST) were based on ST data with GrapeTree^6^. The information on the allelic loci of the cgMLST was based on the profiles of 1,553 coding loci in the EnteroBase *Yersinia* cgMLST scheme (cgMLST V1+HierCC V1). The allelic difference matrix of the isolates was obtained by cgmlst-dists software v0.4.0 based on the allelic locus information.

### Prediction of the phenotypic drug resistance pattern based on whole-genome sequencing

Genes conferring AMR genes were aligned and annotated with the Resistance Gene Identifier (RGI) v5.2.1 of the Comprehensive Antibiotic Resistance Database (CARD) (identity ≧ 80%, coverage ≧ 80%) and ResFinder v4.1 (identity ≧ 80%, coverage ≧ 80%)^7^. Plasmid replicons were detected using PlasmidFinder v2.0 (identity ≧ 80%)^8^. A custom search library was created using the makeblastdb parameter in ncbi-blast v2.11.0+ software to extract DNA sequences of AMR genes and analyze nucleotide mutations via python scripts. The position of AMR genes and plasmids was inferred from the scaffold of the assembly in which they were annotated.

### Statistical and computational analysis

Correlations between phenotypes and genotypes are indicated by sensitivity, specificity, positive predictive value, and negative predictive value. Sensitivity, or true positive rate, quantifies how well a test identifies true positives. Specificity, or true negative rate, quantifies how well a test identifies true negatives. Positive predictive value (PPV) and negative predictive value (NPV) reflect the proportion of positive and negative results that are true positives and true negatives, respectively. The calculation formula is as follows (Monaghan et al., 2021). In this study, the true positives indicated that the isolate had a resistance gene to an antimicrobial agent and exhibited the resistance phenotype to that agent. The true negative indicated that the isolate had no resistance gene to an antimicrobial agent and showed either the sensitive or intermediate resistance phenotype to that agent.


$$S\,e\,n\,s\,i\,t\,i\,v\,i\,t\,y=\frac{T\,r\,u\,e\,P\,o\,s\,i\,t\,i\,v\,e\,s}{T\,r\,u\,e\,P\,o\,s\,i\,t\,i\,v\,s+F\,a\,l\,s\,e\,N\,e\,g\,a\,t\,i\,v\,e\,s};$$

$$S\,p\,e\,c\,i\,f\,i\,c\,i\,t\,y=\frac{T\,r\,u\,e\,P\,o\,s\,i\,t\,i\,v\,e\,s}{T\,r\,u\,e\,P\,o\,s\,i\,t\,i\,v\,e\,s+F\,a\,l\,s\,e\,N\,e\,g\,a\,t\,i\,v\,e\,s};$$

$$P\,o\,s\,i\,t\,i\,v\,e\,P\,r\,e\,d\,i\,c\,t\,i\,v\,e\,V\,a\,l\,u\,e=\frac{T\,r\,u\,e\,P\,o\,s\,i\,t\,i\,v\,e\,s}{T\,u\,r\,e\,P\,o\,s\,i\,t\,i\,v\,e\,s+F\,a\,l\,s\,e\,P\,o\,s\,i\,t\,i\,v\,e\,s};$$

$$N\,e\,g\,a\,t\,i\,v\,e\,P\,r\,e\,d\,i\,c\,t\,i\,v\,e\,V\,a\,l\,u\,e=\frac{T\,r\,u\,e\,N\,e\,g\,a\,t\,i\,v\,e\,s}{T\,r\,u\,e\,N\,e\,g\,a\,t\,i\,v\,e\,s+F\,a\,l\,s\,e\,N\,e\,g\,a\,t\,i\,v\,e\,s}$$

### Nucleotide sequence accession numbers

This whole-genome project was deposited in GenBank under the accession number PRJNA773921. The raw sequence reads were deposited in the Sequence Read Archive (SRA) under accession nos. SRR16693328–SRR16693604.

## Results

### Review of the historical data for isolates

In total, 189 *Y. enterocolitica* isolates were analyzed from 2007 to 2019. Of these, 152 (80.42%) were of animal origin, 24 (12.70%) were obtained from food, and 13 (6.88%) were of patient origin. Animal hosts included pigs (*n* = 120), sheep (*n* = 18), rats (*n* = 5), cattle (*n* = 6), and chickens (*n* = 3). Food samples were derived from meat products, consisting of beef (*n* = 11), pork (*n* = 4), chicken (*n* = 6), and lamb (*n* = 3). Human samples (*n* = 13) were of fecal origin, the majority of which were obtained from children (*n* = 9) (Supplementary Table 3).

Based on traditional phenotypic methods, 183/189 (96.83%) isolates were serotyped. The most common serotypes were O:3 (*n* = 93), O:5 (*n* = 60), O:8 (*n* = 22), and O:9 (*n* = 5). In total, six isolates were reported as O: unidentifiable because the O-antigen did not react with any of the antisera (Table 2). The observed biotypes of *Y. enterocolitica* included biotype 1A (*n* = 92), biotype 2 (*n* = 3), biotype 3 (*n* = 4), biotype 4 (*n* = 83), and biotype 5 (*n* = 7). Biotype 1B was absent (Supplementary Table 4).

**TABLE 2:** Biotype and serotype of 189 *Y. enterocolitica* isolates.

Biotype	O serotype							
	O:3	O:5	O:8	O:9	O:53	O:1,2,5	O:5,8,9	O: unidentifiable
2	0	0	0	3	0	0	0	0
3	4	0	0	0	0	0	0	0
4	82	0	0	0	0	0	0	1
5	7	0	0	0	0	0	0	0
1A	0	60	22	2	1	1	1	5

### Sequence types and core genome multilocus sequence types determination

**FIGURE 1:**
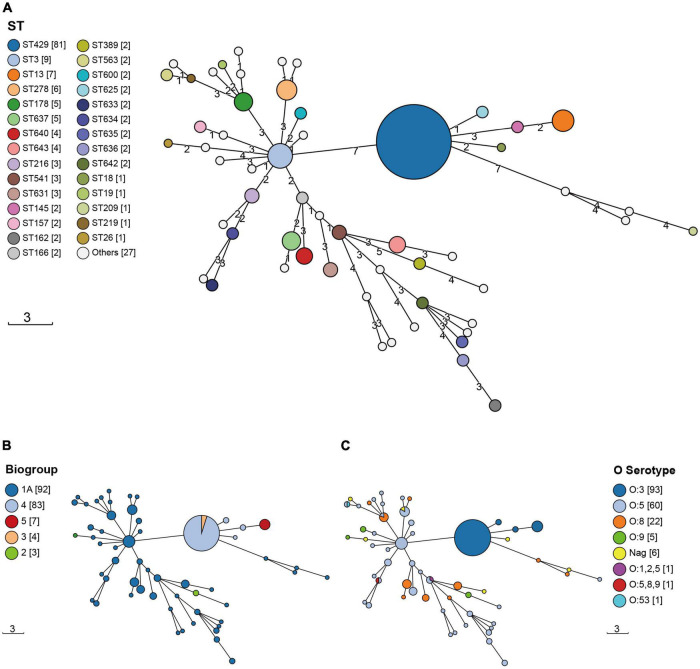
The Minimum Spanning Trees (MST) of *Y. enterocolitica* isolates. STs were identified by the information on the allelic loci of *aar*F, *dfp*, *gal*R, *gln*S, *hem*A, *rfa*E, and *spe*A. **(A)** ST; **(B)** biotype; **(C)** serotype. The circle size was proportional to the number of isolates. Links between circles were represented according to the number of allelic differences between STs.

**FIGURE 2:**
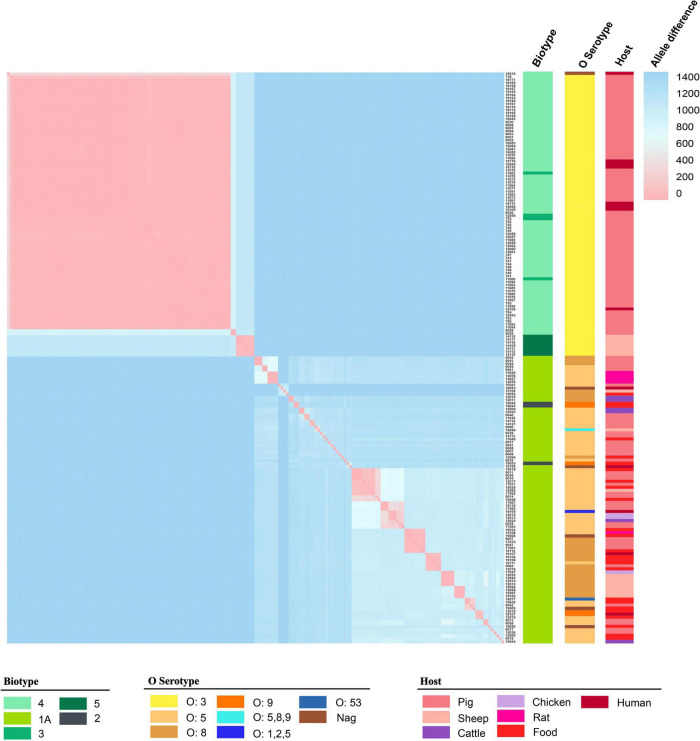
The matrix of allele difference loci of strain. Both the X and Y axes indicated the sample name. Metadata for biotype, serotype, and host were presented.

The 189 isolates were identified as 56 STs and 127 CTs. The most common STs were ST429 at 42.86% (81/189); ST3 at 4.76% (9/189); ST13 at 3.70% (7/189); ST278 at 3.17% (6/189); and ST178 and ST637 at 2.65% (5/189) (Figure 1 and Supplementary Table 4). All of the ST429 isolates were biotypes 3 and 4, with serotype O:3 and the hosts were pigs and humans. All of the ST13 isolates were biotype 5, with serotype O:3 and the hosts were sheep. The serotypes of biotype 1A isolates were mainly O:5, O:8, and O:9, and the hosts were pigs, food, sheep, chickens, cattle, humans, and rats. 127 CTs were divided into 62 genetic clusters sharing 100 cgMLST alleles as a threshold, linked by single-stranded clustering criteria. Denoted as HC100 (Hierarchical clustering) in EnteroBase, indicating that the clusters include all strains with no more than 100 alleles linked. CgMLST analysis revealed the core genome diversity of strains with the same ST from 0 to 84 allelic differences (Figure 2). Of these, the pathogenic isolates comprised 5 STs and 50 CTs, and the isolate of biotype 1A contained 51 STs and 77 CTs. This suggested that biotype 1A was significantly more polymorphic compared to biotypes 3, 4, and 5.

### Antimicrobial susceptibility testing

The isolates were tested using conventional microbiology methods with a panel of 13 antimicrobial agents (Supplementary Table 5). The highest resistance level was 97.88% for CZO, followed by AMP (44.97%), CIP (25.40%), STR (11.11%), and TET (10.58%). Among the isolates from Ningxia, 27.51% (*n* = 52) were MDR strains. Of these, 61.54% (*n* = 32) of the isolates were resistant to three antimicrobial agents, with the most common resistance spectrum being AMP-CZO-CIP (*n* = 21). In addition, 26.92% (*n* = 14) of the isolates were resistant to four antimicrobial agents, with the most frequent resistance spectrum being AMP-CZO-SXT-CIP (*n* = 6). The resistance spectrum of the three isolates to five antimicrobial agents was AMP-TET-CZO-CIP-CHL (NX18083) and AMP-CZO-SXT-CIP-STR (NX18118). Two isolates were resistant to six antimicrobial agents, with a resistance spectrum of AMP-TET-CZO-SXT-CIP-STR (NX18084) and AMP-TET-CZO-CIP-STR-CHL (NX18108), respectively. One isolate (NX18109) was resistant to seven antimicrobial agents and had a resistance spectrum of AMP-TET-CZO-SXT-CIP-STR-CHL. Another isolate (NX18110) was resistant to eight antimicrobial agents and had a resistance spectrum of AMP-TET-CZO-SXT-CIP-GEN-STR-CHL.

Isolates from different host sources showed statistically significant differences in resistance rates/intermediate resistance rates to TET, CXM, SXT, CIP, STR, and CHL. Isolates from different hosts showed the same rates of resistance to CZO, POL, and IPM. Since only one isolate was resistant to GEN, resistance to GEN was not discussed. The resistance rates/intermediate resistance rates of isolates from different host sources to AMP, CZO, CAZ, and STR showed the highest rates for isolates from patient sources, followed by those from animals, and the lowest rates for those from food sources. Isolates of animal origin had the highest resistance rates to SAM. Food-derived isolates showed higher rates of resistance to CHL than animal-derived ones (Table 3). With regards to the pathogenicity of the isolates, pathogenic (biotypes 2, 3, 4, and 5) and non-pathogenic (biotype 1A) isolates showed statistically significant differences in resistance rates to TET, CIP, and STR. While SXT resistance rates did not apply to the χ^2^ test, it was very evident in Table 4 that resistance rates to SXT were higher for pathogenic isolates compared to non-pathogenic isolates.

**TABLE 3:** Resistance rates of isolates from different sources.

Antimicrobial agents		Animal (*n* = 152)						Food (*n* = 24)	Human (*n* = 13)
		Pig (*n* = 120)	Sheep (*n* = 18)	Cattle (*n* = 6)	Rat (*n* = 5)	Chicken(*n* = 3)	Total (n = 152)		
AMP	S (%)	8.33	0	0	0	66.67	7.89	4.17	7.70
	I (%)	40.83	83.33	50	40	33.33	46.05	62.5	38.46
	R (%)	50.83	16.67	50	60	0	46.05*^a^*	33.33*^a^*	53.85*^a^*
SAM	S (%)	65.83	88.89	66.67	40	100	68.42	83.33	53.85
	I (%)	32.5	5.56	33.33	60	0	29.61	16.67	46.15
	R (%)	1.67	5.56	0	0	0	1.97	0	0
TET	S (%)	85.83	100	100	100	100	88.82	91.67	53.85
	I (%)	3.33	0	0	0	0	2.63	4.17	0
	R (%)	10.83	0	0	0	0	8.55*^a^*	4.17*^a^*	46.15*^b^*
CZO	S (%)	3.33	0	0	0	0	2.63	0	0
	I (%)	0	0	0	0	33.33	0.66	0	7.70
	R (%)	96.67	100	100	100	66.67	96.71*^a^*	100*^a^*	92.30*^a^*
CXM	S (%)	99.17	94.44	100	100	100	98.68	100	76.92
	I (%)	0.83	5.56	0	0	0	1.32*^a^*	0	23.08*^b^*
CAZ	S (%)	100	100	100	100	100	100	100	76.92
	I (%)	0	0	0	0	0	0	0	23.08
SXT	S (%)	94.17	100	100	100	100	95.39	100	69.23
	R (%)	5.83	0	0	0	0	4.61*^a^*	0	30.77*^b^*
POL	I (%)	100	100	100	100	100	100	100	100
CIP	S (%)	54.17	100	83.33	100	100	63.16	75	38.46
	I (%)	12.5	0	0	0	0	9.87	20.83	15.38
	R (%)	33.33	0	16.67	0	0	26.97*^a^*	4.17*^b^*	46.15*^a^*
GEN	S (%)	100	100	100	100	100	100	100	92.30
	R (%)	0	0	0	0	0	0	0	7.70
STR	S (%)	88.33	100	100	100	100	90.79	91.67	61.54
	R (%)	11.67	0	0	0	0	9.21*^a^*	8.33*^a^*	38.46*^b^*
CHL	S (%)	100	100	100	100	100	100	91.67	61.54
	R (%)	0	0	0	0	0	0	8.33*^a^*	38.46*^b^*
IPM	S (%)	100	100	100	100	100	100	100	100

AMP, ampicillin; SAM, ampicillin/sulbactam, TET, tetracycline; CZO, cefazolin; CXM, cefuroxime; CAZ, ceftazidime; SXT, trimethoprim/sulfamethoxazole; POL, polymyxin; CIP, ciprofloxacin; GEN, gentamicin; STR, streptomycin; CHL, chloramphenicol; IPM, imipenem; I, intermediate resistant; S, susceptibility. Different superscript lowercase letters indicate significant differences (p < 0.05). The same superscript lowercase letters indicate no statistical difference. No superscript letters indicate not applicable.

**TABLE 4:** Resistance rates of pathogenic and non-pathogenic isolates.

Antimicrobial agents		Pathogenic (n = 96)					Non-pathogenic (*n* = 87)
		4/O:3 (*n* = 82)	3/O:3 (*n* = 4)	5/O:3 (*n* = 7)	2/O:9 (*n* = 3)	Total (*n* = 96)	
AMP	S	9	0	0	0	9	3
	I	31	2	7	2	42	45
	R	42	2	0	1	45*^a^*	39*^a^*
SAM	S	55	3	7	2	67	61
	I	26	1	0	1	28	25
	R	1	0	0	0	1*^a^*	1*^a^*
TET	S	64	4	7	3	78	81
	I	1	0	0	0	1	3
	R	17	0	0	0	17*^a^*	3*^b^*
CZO	S	3	1	0	0	4	0
	I	0	0	0	0	0	1
	R	79	3	7	3	92*^a^*	86*^a^*
CXM	S	79	4	7	3	93	86
	I	3	0	0	0	3	1
CAZ	S	79	4	7	3	93	87
	I	3	0	0	0	3	0
SXT	S	71	4	7	3	85	87
	R	11	0	0	0	11	0
CIP	S	30	1	7	3	41	74
	I	14	0	0	0	14	7
	R	38	3	0	0	41*^a^*	6*^b^*
GEN	S	81	4	7	3	95	87
	R	1	0	0	0	1	0
STR	S	67	3	7	3	80	82
	R	15	1	0	0	16*^a^*	5*^b^*
CHL	S	78	4	7	3	92	85
	R	4	0	0	0	4*^a^*	2*^a^*
POL	I	82	4	7	3	96	87
IPM	S	82	4	7	3	96	87

AMP, ampicillin; SAM, ampicillin/sulbactam, TET, tetracycline; CZO, cefazolin; CXM, cefuroxime; CAZ, ceftazidime; SXT, trimethoprim/sulfamethoxazole; POL, polymyxin; CIP, ciprofloxacin; GEN, gentamicin; STR, streptomycin; CHL, chloramphenicol; IPM, imipenem; R, resistant; I, intermediate resistant; S, susceptibility. Different superscript lowercase letters indicate significant differences (p < 0.05). The same superscript lowercase letters indicate no statistical difference. No superscript letters indicate not applicable.

### Prediction of phenotypic drug resistance pattern based on whole-genome sequencing

**FIGURE 3:**
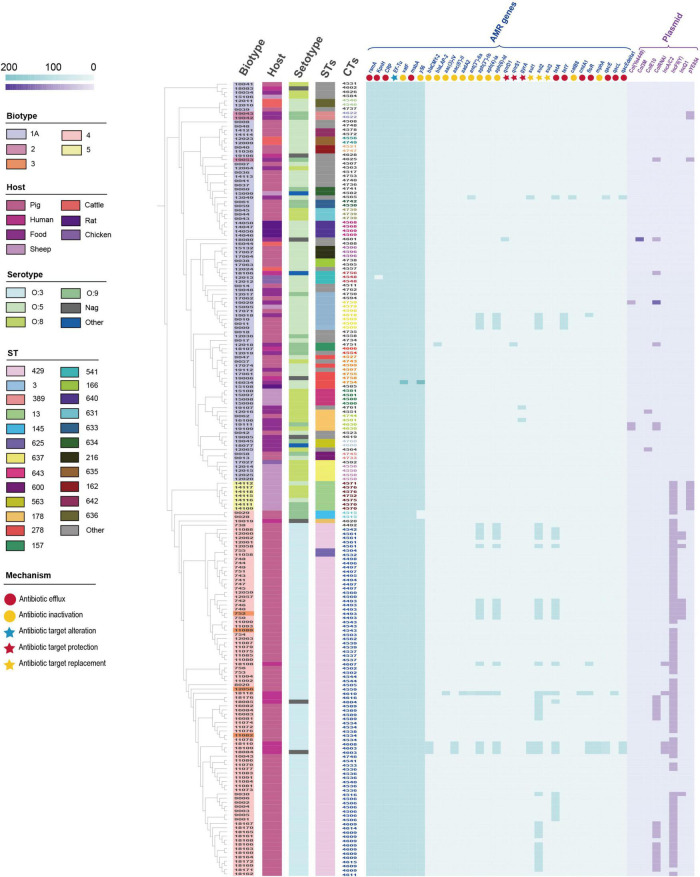
Distribution of antimicrobial resistance (AMR) genes and plasmids in 189 *Y. enterocolitica* strains. Metadata for biotype, host, serotype, STs, CTs, and resistance mechanism were presented. The 20 HC100 in different colors indicated 20 genetic clusters with a threshold of sharing 100 cgMLST alleles, linked by single-stranded clustering criteria. Gray CTs indicated the other 42 HC100.

Using the Comprehensive Antibiotic Resistance Database (CARD) and ResFinder, 189 isolates were annotated to 32 AMR genes in 10 classes (Figure 3 and Supplementary Table 6). Mechanisms of resistance included antibiotic efflux, antibiotic target alteration, antibiotic target protection, antibiotic target replacement, and antibiotic inactivation. Antibiotic efflux and antibiotic inactivation accounted for 78.13% of AMR genes (*n* = 25). AMR genes, including *rsm*A, CRP, KpnH, EF-Tu, *msb*A, *vat*F, and *y*56, were annotated in 98.94–100% of the isolates. The pathogenic isolates (biotype 2, 3, 4, and 5) were annotated with 71.88% (*n* = 23) of the resistance genes, while the non-pathogenic isolates (biotype 1A) were annotated with 78.13% (*n* = 25) of the resistance genes. Excluding the seven resistance genes (*rsm*A, CRP, KpnH, EF-Tu, *msb*A, *vat*F, and *y*56), 41.49% (*n* = 39) of the pathogenic isolates had the remaining 19 resistance genes; whereas 4.26% (*n* = 4) of the non-pathogenic isolates had the other 17 resistance genes.

In addition, 8 plasmids were annotated in 189 isolates (Figure 3 and Supplementary Table 6). Plasmids were found in 89.69% (*n* = 87) of the pathogenic isolates and 11.96% (*n* = 11) of the non-pathogenic isolates. Plasmid IncFII(Y) (*n* = 89) and ColRNAI (*n* = 24) were the predominant plasmid type. Plasmids IncA/C (*n* = 5), IncFII(Y), IcnQ1 (*n* = 9), and pYE854 (*n* = 10) were found in pathogenic isolates. In contrast, Col(Ye4449) (*n* = 3), Col3M (*n* = 1), and ColE10 (*n* = 2) were present in non-pathogenic isolates. Furthermore, we inferred that several samples of the IncQ1 plasmid carried the *aph*(6)-Id, *aph*(3″)-Ib, and *sul*2 genes by locating the AMR gene and plasmid in the scaffold (Supplementary Figure 1).

The number and variety of resistance genes and plasmids were significantly higher in the pathogenic isolates than those in the non-pathogenic isolates. AMR genes were mainly concentrated in second-generation cephalosporin resistance, aminoglycoside resistance, sulfonamide resistance, tetracycline resistance, and chloramphenicol resistance. The primary plasmid types were IncFII(Y) and ColRNAI. Compared to isolates of biotypes 1A, isolates of biotypes 2, 3, 4, and 5 had a higher tendency to be resistant and were more likely to be MDR strains.

### Genotype–phenotype comparisons

Genotypes were compared with phenotypic resistance to 11 clinically relevant antimicrobial agents (AMP, TET, CZO, CAZ, CXM, SXT, CIP, GEN, STR, CHL, and IPM). In clinical practice, these antibacterial agents are commonly applied in the treatment of intestinal infections. The genotypic and phenotypic correlations were shown in Table 5. Sensitivity indicated the probability that the isolate had a resistance gene when it presented the resistance phenotype. Specificity showed the probability that the isolate had no resistance genes when it displayed the non-resistant phenotype (sensitive or intermediate resistance). The sensitivity of *sul*2/CIP was 100%. The genes of *aad*A2, *aph*(3″)-Ib, *aph*(6)-Id, and *aac*(3)-IVa were all 100% sensitive to GEN. The sensitivity of *y*56/CZO was 98.91%. The genes of *aph*(3″)-Ib and *aph*(6) were 90.48% sensitive to STR. The PPV represented the percentage of isolates that actually had resistance genes when they presented the resistance phenotype. The NPV was the percentage of isolates presenting the sensitive or intermediate resistant phenotype without the resistance gene. The PPV of *bla*_*CMY2*_/CZO, *bla*_*LAP*–2_/CZO, *tet*(Y)/TET, and *sul*3/SXT was 100%. The PPV of *aad*A2/GEN+STR, *aac*(3)-IVa/GEN+STR was 100%. The PPV of *aph*(3″)-Ib, *aph*(4)-Ia/STR, *aph*(6)-Id, and *aac*(6′)-Il genes to STR was 100%. *Cat*B8 and *cml*A1 genes also had the 100% PPV for CHL. The *y*56 gene had 100% NPV for AMP, CAZ, and CXM. The genes of *aad*A2, *aph*(3″)-Ib, *aph*(6)-Id, and *aac*(3)-IVa had 100% NPV to GEN.

**TABLE 5:** The genotypic and phenotypic correlations of the AMR genes.

Category	AMR gene	Sensitivity (%)	Specificity (%)	PPV (%)	NPV (%)	Note
β-lactams	*y*56	49.43	69.23	95.60	9.18%	AMP
		0	1.06	0	100	IPM
Cephalosporins	*y*56	98.91	0	96.79	0	CZO
		0	1.06%	0	100	CAZ
		0	1.06%	0	100	CXM
	*bla*_*CMY2*_	1.64	100	100	3.23	CZO
		0	100	0	3.23	CAZ
		0	100	0	3.23	CXM
	*bla*_*LAP–2*_	0.55	100	100	3.19	CZO
		0	99.47	0	100	CAZ
		0	99.47	0	100	CXM
Tetracyclines	*tet*(A)	76.92	97.55	83.33	96.36	
	*tet*(Y)	15.38	100	100	88.11	
Trimethoprim/sulfamethoxazole	*sul*1	9.09	97.75	20	94.57	
	*sul*2	100	84.83	28.95	100	
	*sul*3	9.09	100	100	94.68	
Fluoroquinolones	QnrD1	0	99.15	0	62.23	
	QnrS1	0	99.15	0	62.23	
Aminoglycosides	*aad*A2	100	100	100	100	GEN
		4.76	100	100	89.36	STR
	*aph*(3″)-Ib	100	89.89	5	100	GEN
		90.48	100	100	98.82	STR
	*aph*(4)-Ia	0	99.47	0	99.47	GEN
		4.76	100	100	89.36	STR
	*aph*(6)-Id	100	89.89	5	100	GEN
		90.48	100	100	98.82	STR
	*aac*(3)-IVa	100	100	100	100	GEN
		4.76	100	100	89.36	STR
	*aac*(6’)-Il	0	98.40	0	99.46	GEN
		14.29	100	100	90.32	STR
Chloramphenicols	*cat*B8	14.29	100	100	96.81	
	*cml*A1	14.29	100	100	96.81	
	*flo*R	85.71	99.45	85.71	99.45	

AMP, ampicillin; CZO, cefazolin; CXM, cefuroxime; CAZ, ceftazidime; GEN, gentamicin; STR, streptomycin; IPM, imipenem; PPV, Positive predictive Value; NPV, negative predictive value.

Additionally, we analyzed the mutation loci of the AMR genes. The *y*56 gene was present in 98.94% of the isolates in this study. The percentage of isolates showing both intermediate resistance and resistance to ampicillin was 46.05% (Table 3). Two isolates (NX09028 and NX09029) were absent of the *y*56 gene and showed ampicillin sensitivity (Figure 3 and Supplementary Table 6). However, it was noteworthy that 11 isolates showed ampicillin sensitivity although the *y*56 gene was presented. We analyzed the DNA sequences of the y56 gene, including ampicillin-susceptible, intermediate resistant, and resistant strains, and the results indicated that the order of DNA alignment was closely related to the biotype but not the resistance phenotype (Supplementary Figure 2).

Moreover, to address fluoroquinolone resistance, we analyzed *gyr*A and *par*C, two key genes that contribute to fluoroquinolone resistance. Among the 48 fluoroquinolone resistant isolates, the *gyr*A gene included seven mutations: Phe50→Tyr, Ser83→Ile, Ala336→Gly, Gln512→Leu, Ala828→Thr, Asp847→Asn, and Thr878→Ala. The *par*C gene had a Pro643→Ser mutation (Supplementary Figure 2). Mutations affecting the efficacy of DNA gyrase primarily appear in Ala67-Gln106 of the GyrA protein, a region known as the quinolone drug resistance determining region (QRDR) (Robab et al., 2020). The Ser83 amino acid in this region plays a critical role in the quinolone-enzyme interaction, and it is the most significant mutation leading to quinolone resistance (Correia et al., 2017).

### Analysis of drug resistance in pig-derived strains

**FIGURE 4:**
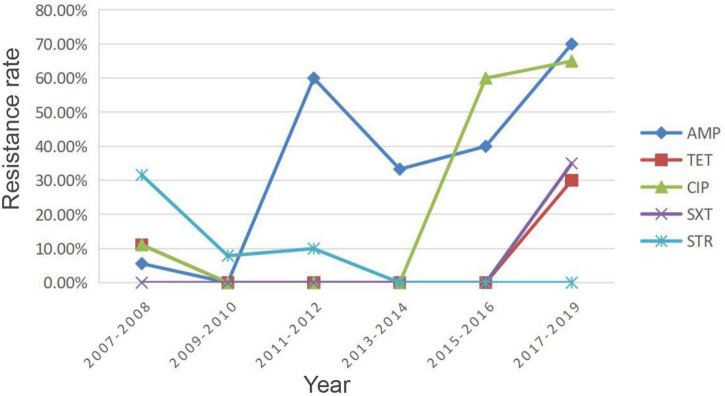
Resistance rates of pig-derived strains from 2007 to 2019. AMP, ampicillin; TET, tetracycline; SXT, trimethoprim/sulfamethoxazole; CIP, ciprofloxacin; STR, streptomycin.

Considering the continuity of the samples and the homogeneity of the sample size across years, we counted the resistance rates of pig-sourced samples between 2007 and 2019 in 2-year intervals (Figure 4). Resistance of the isolates to AMP, TET, and CIP was low during 2007–2008, and the resistance rate dropped to zero by 2010. From 2009 to 2016, isolates were sensitive to TET, and between 2017 and 2019, the rate of resistance to TET increased by 30%. In 2009, the resistance rate to CIP was 0; it increased to 60% by 2016 and has continued to rise. The resistance rate of isolates to AMP increased substantially in 2011 and, after a decrease in 2014, increased to 70% in 2018. Resistance of isolates to SXT, which had been sensitive for the first decade, began increasing in 2016 up to 30% in 2019. In contrast to the above was STR, the resistance rate was 30% in 2007 and gradually decreased thereafter to 0 in 2013, continuing until 2019.

## Discussion

The current study aimed to investigate factors affecting diversity and resistance in *Y. enterocolitica* isolates from Ningxia and to compare the predicted resistance patterns from WGS data with phenotypic results from classical microbial assays. *Y. enterocolitica* produces β-lactamases and is therefore naturally resistant to AMP, amoxicillin, and first-generation cephalosporins (Fredriksson-Ahomaa et al., 2012). This is consistent with the resistance and intermediate resistance rates of AMP reported in this study. The intrinsic resistance to these β-lactam antibiotics is due to the production of two chromosomally encoded beta-lactamase genes *bla*A and *bla*B (Fàbrega and Vila, 2012). *Bla*A encodes for one class A enzyme showing constitutive expressions and is involved in limiting the susceptibility to penicillins and cephalosporins. *Bla*B encodes for an inducible class C enzyme (AmpC-type) which is associated with cephalosporin susceptibility (Bent and Young, 2010). In this study, the *y*56 gene also called the *bla*A-2 gene (GenBank: AY954728.1), contributed significantly to intermediate resistance and resistance to AMP. This was consistent with the findings of Bent and Young (2010). Furthermore, Stock et al. (1999) demonstrated that the susceptibility of *Y. enterocolitica* to β-lactam antibiotics was associated with different biotypes. In contrast, there was no statistical difference in susceptibility to ampicillin between pathogenic isolates (biotypes 2, 3, 4, and 5) and non-pathogenic isolates (biotype 1A) in this study. It is speculated that the reason for the difference may be that the strains of biotypes 2, 4, and 5 in Stock’s study contained both *bla*A and *bla*B genes and that the expression of these two genes was differentially regulated between the biotypes. In contrast, the isolates in this study contained only the *bla*A gene. While the 11 AMP-sensitive isolates may be related to the regulation and expression of β-lactamase.

In addition, the isolates showed 25.40, 11.11, and 10.58% resistance to CIP, STR, and TET, respectively, indicating that the isolates were not highly resistant to these two antimicrobial agents. The isolates showed low resistance to CHL and SXT, with resistance rates of only 3.70 and 5.82%, respectively. These results are in partial agreement with the findings of Sun et al. (2013); Ye et al. (2015), and Verbikova et al. (2018). Variation in resistance levels of *Y. enterocolitica* worldwide may be caused by regional and temporal differences, as well as isolates from different sources. Another reason for the discrepancies could be due to obtaining false positive or false negative results from the tests used for identification (both genotypic and phenotypic).

A study by Baumgartner et al. (2007) identified the existence of MDR “super” strains. The same finding was confirmed in the present study, such as NX18084, NX18108, and NX18110. We calculated the trends in overall resistance and intermediary rates for AMP, TET, SXT, CIP, and STR in isolates of pig origin from 2007 to 2019, as shown in Figure 4. Owing to the characteristics of *Y. enterocolitica*, resistance rates of AMP remained at high levels. The resistance rates of TET, SXT, and CIP showed an overall increasing trend, although there were some decreases. There is reasonable suspicion that the origin of the strain is correlated with exposure to antibiotics. These results suggest that careful observation of changes and trends in the AMR of *Y. enterocolitica* isolates is needed due to the long-term use of antimicrobial agents in the farming industry. Conversely, there was an overall downward trend in STR resistance rates. STR is an important antibiotic for humans and livestock. As a veterinary antibiotic, it is frequently used as a feed supplement to treat acute infections caused by a variety of sensitive bacteria (Li, 2017). The decline in STR resistance rates was a further indication that the breeding industry in the Ningxia region had become regulated after years of supervision and management.

In addition to AMR genes, the horizontal transfer of plasmids containing resistance genes is an essential element for the dispersion of antimicrobial resistance (Butaye et al., 2014). IncFII showed high occurrence in resistant plasmids, they were detected in bacteria from different countries and different origins, and their appearance seems to be closely linked to positive selection exerted by antimicrobial use (Carattoli, 2013). IncF plasmids carry different replication subtypes (e.g., FIA, FIB, and FII), most of which were associated with extended-spectrum β-lactamases (ESBLs) (Carattoli, 2013). Their great intracellular diversity and rapid evolution of their replicon regulatory sequences enabled them to spread successfully through the *Enterobacteriaceae* (Villa et al., 2010). Notably, the TET, STR, and SXT resistance exhibited by the isolates in this study were mediated by the *tet*A, *aph*(6)-Id, *aph*(3″)-Ib, and *sul*2 genes located on the IncQ1 plasmid. IncQ plasmid derivatives are not self-transmissible by conjugation and can be mobilized at high frequencies in the presence of helper plasmids (Carattoli, 2009). Compared to IncF plasmids, IncQ is able to replicate over a wider host range (Lawley et al., 2004). IncF plasmids are restricted by the host range to the genera of *Enterobacteriaceae* (Lawley et al., 2004). Three strategies have emerged for IncQ plasmids to obtain a broad host range, the multifunctionality of plasmid replication, the self-sufficiency of encoded proteins required to establish replisomes after splicing, and the presence of two or more multifunctional replicons on the same plasmid, limiting incompatibility effects (Toukdarian, 2004).

As can be seen from the MST in Figure 1, the distribution of AMR isolates was predominantly clustered in 4/O:3. Our previous study on the molecular characterization of *Y. enterocolitica* in Ningxia (unpublished) showed that STs and CTs of isolates from Ningxia were geographically specific. In addition to β-lactam resistance, AMR included TET resistance, SXT resistance, CIP resistance, STR resistance, and CHL resistance. Karlsson et al. (2021) showed that an outbreak caused by *Y. enterocolitica* 4/O:3 in fresh prewashed spinach occurred in Sweden in 2019. These strains had a multidrug-resistant molecular signature, including AMP, CHL, SXT, TET, STR, and erythromycin (ERY). It is known that biotype 1A is non-pathogenic, and biotypes 2, 3, 4, and 5 are low pathogenic (Sun et al., 2013). Figure 3 and Table 4 show that the pathogenic isolates were more likely to show a resistant phenotype than the non-pathogenic isolates. In many respects, genetically encoded antimicrobial resistance can be considered a subtype of virulence factors since it promotes host pathogenesis and contributes to persistent or chronic disease (Schroeder et al., 2017). Evidently, antibiotics exert selective pressure on bacteria, accelerating evolution and allowing simultaneous genetic and metabolic adjustments to produce AMR or MDR phenotypes (Vranakis et al., 2014). Increased virulence may naturally evolve in response to or concurrently with increased antimicrobial resistance.

Resistance rates to many antibiotics showed significant differences between strains of different origins, with strains of patient origin, in particular, having higher resistance rates and being more likely to develop MDR, as the clinical application of antibiotics has generally expanded the AMR gene pool in the gut. Since *Y. enterocolitica* infections in humans most often originate from pigs and improperly prepared contaminated food, this was also reflected in this study. Pig-derived isolates, food-derived isolates, and patient-derived isolates had the same HC100 and the same drug sensitivity pattern, suggesting that the strains were likely to be transmitted from pigs or food to humans. These strains may have been selected over a longer period of time in host pigs and have been transmitted and found to a large extent in both humans and pigs. The relatively small number of patients in this study limits its scope. One reason for the small sample sizes is that the Ningxia Hui Autonomous Region is an area with a high Muslim population, and pig farms are relatively concentrated and usually located outside of Muslim settlements. Combined with the high proportion of halal diets, this resulted in a low number of fecal samples from patients with diarrhea. Another reason is that research into yersiniosis has been neglected for many years. The United Kingdom Standards for Microbiology Investigations of Fecal Specimens for Enteric Pathogens state that testing of fecal specimens for *Yersinia* species is only recommended when clinical suspicion has been raised (Public Health England, 2014). Surveillance of the disease has ceased in China for the last 20 years, resulting in *Y. enterocolitica* infection rarely being considered by clinicians in the diagnosis of gastrointestinal disease and testing staff rarely provide a basis for accurate diagnosis (Guo et al., 2011).

To compare phenotypes and genotypes of AMR genes, the statistical principles of sensitivity, specificity, positive predictive value, and negative predictive value were applied. They are usually used to describe the usefulness of diagnostic tests (Akobeng, 2007). Sensitivity and specificity are important measures of the diagnostic accuracy of the tests (Monaghan et al., 2021). The two are inversely proportional, meaning that the higher the sensitivity, the lower the specificity and vice versa. However, in practical cases, rather than knowing the proportion of isolates that carry a certain resistance gene and display resistance, it frequently makes more sense to predict whether an isolate is resistant or not based on the presence or absence of the resistance gene. The PPV and NPV reflect the proportion of positive and negative outcomes for true positives and true negatives, respectively (Monaghan et al., 2021). The PPV answers the question: if a certain resistance gene is present, is the likelihood of showing resistance? Conversely, the NPV answers the question: if there is no resistance gene, the probability of displaying non-resistance? For example, for CIP resistance, all of the isolates were annotated to the fluoroquinolone-resistant efflux pump CRP and *rsm*A. The efflux pump is the principal mechanism of intrinsic and acquired AMR in clinical infections (Azargun et al., 2020). It has substrate specificity and can excrete a variety of toxic compounds from bacterial cells, causing MDR. However, only 25.40% (48/189) of the isolates showed a CIP-resistant phenotype. The PPV and NPV of the QnrS1 and QnrD1 genes to CIP were 0 and 62.23%, respectively. Through analysis of the genes *gyr*A and *par*C, it was found that mutations in these two genes were the primary factors responsible for CIP resistance. Only 11 of the 13 antibacterial agents were analyzed, as SAM (ampicillin/sulbactam) is a β-lactamase inhibitor. After the combination of ampicillin and sulbactam, the resistance rate of the isolates to AMP decreased from 44.97% (85/189) to 1.58% (3/189), indicating that sulbactam was able to effectively inhibit the activity of β-lactamase. In addition, POL is a peptide antibiotic. Among the annotated AMR genes, only the efflux pump KpnH gene acted against the peptide antibiotics, but the resistance effect was non-specific. Moreover, all isolates in this study showed intermediate resistance to POL, so they were not analyzed here.

A possible explanation for the phenotypic and genotypic incongruity lies in the complexity of the AMR mechanisms in *Y. enterocolitica*. The pattern and level of expression of AMR genes, the interaction between multiple AMR genes, and the formation of biofilms may all influence the resistance characteristics of strains (Blair et al., 2015; Chang et al., 2015). An additional cause for discrepancies is linked to the use of Critical Concentrations (CCs) established at excessively high values that do not represent the true distribution of the wild-type and mutant bacterial population (Cirillo et al., 2017). The CCs is generally determined as an epidemiological cut-off value (ECOFF) or one dilution higher than it. For most drugs, the ECOFF separates wild-type strains expected to be sensitive from those expected to be resistant to a selected drug. If drugs can be administered at higher doses without a high risk of toxicity, concentrations above CC can be tested to predict susceptibility to treatment when higher doses of the drug are used to achieve higher plasmatic concentrations. In this case, a “clinical breakpoint” can be established (Cirillo et al., 2017). Furthermore, these discrepancies may be due to unidentified resistance genes. This points to a limitation of WGS, as it only detects previously identified genes. With new resistance genes being discovered and added to the database, the number of strains expressing phenotypic resistance but for which no gene is found should be reduced, thereby increasing the sensitivity of the assay.

Our study had several limitations. First, the sample size from patient sources was small and was only collected in 2018 and 2019. The reason is that symptoms of diarrhea caused by *Y. enterocolitica* tend to be mild and most cases are self-limiting. Patients rarely visit outpatient clinics. Secondly, there is a lack of quantitative homogeneity and temporal consistency in the collection of samples from both food sources and patient sources. This could potentially introduce bias into the analysis of drug resistance trends. Finally, there is a lack of epidemiologically relevant information on isolates from diarrhea patients. Investigation of related strains from human, animal, and food sources might clarify the distribution pattern of *Y. enterocolitica* drug resistance in different hosts.

## Data availability statement

The datasets presented in this study can be found in online repositories. The names of the repository/repositories and accession number(s) can be found below: https://www.ncbi.nlm.nih.gov/bioproject/PRJNA773921.

## Ethics statement

The animal study was reviewed and approved by the Research Animal Ethics and Ethical Committee of the Chinese Centre for Disease Control and Prevention.

## Author contributions

MS, XL, and QH isolated and identified the isolates. YY wrote the main manuscript text. YY, YK, and SX analyzed the data and prepared Figures 1–4. YY, YC, FL, and SC conducted antimicrobial resistance tests. QH, Z-JL, and X-ZZ participated in the design of this study and reviewed the manuscript. Z-JL supported this study. All authors read and approved the final manuscript.

## Conflict of interest

The authors declare that the research was conducted in the absence of any commercial or financial relationships that could be construed as a potential conflict of interest.

## Publisher’s note

All claims expressed in this article are solely those of the authors and do not necessarily represent those of their affiliated organizations, or those of the publisher, the editors and the reviewers. Any product that may be evaluated in this article, or claim that may be made by its manufacturer, is not guaranteed or endorsed by the publisher.

## Footnotes

1
http://soapdenovo2.sourceforge.net/

2
http://bioinf.spbau.ru/spades

3
https://www.bcgsc.ca/resources/software/abyss

4
https://bio.tools/cisa

5
http://enterobase.warwick.ac.uk/species/index/yersinia

6
https://achtman-lab.github.io/GrapeTree

7
https://bitbucket.org/genomicepidemiology/resfinder

8
https://bitbucket.org/genomicepidemiology/plasmidfinder

## References

[B1] Ahmed H. A. Tahoun A. B. M. B. Abou Elez R. M. M. Abd El-Hamid M. I. Abd Ellatif S. S. (2019). Prevalence of *Yersinia enterocolitica* in milk and dairy products and the effects of storage temperatures on survival and virulence gene expression. *Int. Dairy J.* 94 16–21.

[B2] Akobeng A. K. (2007). Understanding diagnostic tests 1: sensitivity, specificity and predictive values. *Acta Paediatr.* 96 338–341. 10.1111/j.1651-2227.2006.00180.x 17407452

[B3] Azargun R. Gholizadeh P. Sadeghi V. Hosainzadegan H. Tarhriz V. Yousef M. M. (2020). Molecular mechanisms associated with quinolone resistance in *Enterobacteriaceae*: review and update. *Trans. R. Soc. Trop. Med. Hyg.* 114 770–781. 10.1093/TRSTMH/TRAA041 32609840

[B4] Baumgartner A. Küffer M. Suter D. Jemmi T. Rohner P. (2007). Antimicrobial resistance of *Yersinia enterocolitica* strains from human patients, pigs and retail pork in Switzerland. *Int. J. Food Microbiol.* 115 110–114. 10.1016/J.IJFOODMICRO.2006.10.008 17196695

[B5] Bent Z. W. Young G. M. (2010). Contribution of BlaA and BlaB beta-lactamases to antibiotic susceptibility of *Yersinia enterocolitica* biovar 1B. *Antimicrob. Agents Chemother.* 54 4000–4002. 10.1128/AAC.01754-09 20547799 PMC2935026

[B6] Blair J. M. A. Webber M. A. Baylay A. J. Ogbolu D. O. Piddock L. J. V. (2015). Molecular mechanisms of antibiotic resistance. *Nat. Rev. Microbiol.* 13 42–51. 25435309 10.1038/nrmicro3380

[B7] Bottone E. J. (1997). *Yersinia enterocolitica*: the charisma continues. *Clin. Microbiol. Rev.* 10 257–276. 10.1128/CMR.10.2.257 9105754 PMC172919

[B8] Butaye P. van Duijkeren E. Prescott J. F. Schwarz S. (2014). Antimicrobial resistance in bacteria from animals and the environment. *Vet. Microbiol.* 171 269–272. 10.1016/J.VETMIC.2014.04.009 24852141

[B9] Carattoli A. (2009). Resistance plasmid families in *Enterobacteriaceae*. *Antimicrob. Agents Chemother.* 53 2227–2238. 10.1128/AAC.01707-08 19307361 PMC2687249

[B10] Carattoli A. (2013). Plasmids and the spread of resistance. *Int. J. Med. Microbiol.* 303 298–304. 10.1016/j.ijmm.2013.02.001 23499304

[B11] Chang H. H. Cohen T. Grad Y. H. Hanage W. P. O’Brien T. F. Lipsitch M. (2015). Origin and proliferation of multiple-drug resistance in bacterial pathogens. *Microbiol. Mol. Biol. Rev.* 79 101–116. 10.1128/MMBR.00039-14 25652543 PMC4402963

[B12] Cirillo D. M. Miotto P. Tortoli E. (2017). Evolution of phenotypic and molecular drug susceptibility testing. *Adv. Exp. Med. Biol.* 1019 221–246. 10.1007/978-3-319-64371-7_12 29116638

[B13] CLSI (2020). *Performance Standards for Antimicrobial Susceptibility Testing; Twenty-Seven Informational Supplement. M100-S30.* Wayne, PA: Clinical and Laboratory Standards Institute.

[B14] Correia S. Poeta P. Hébraud M. Capelo J. L. Igrejas G. (2017). Mechanisms of quinolone action and resistance: where do we stand? *J. Med. Microbiol.* 66 551–559. 10.1099/jmm.0.000475 28504927

[B15] European Food Safety Authority, and European Centre for Disease Prevention and Control (2021). The European Union Summary Report on antimicrobial resistance in zoonotic and indicator bacteria from humans, animals and food in 2018/2019. *EFSA J.* 19:e06490. 10.2903/j.efsa.2021.6490 33868492 PMC8040295

[B16] Fàbrega A. Vila J. (2012). *Yersinia enterocolitica*: pathogenesis, virulence and antimicrobial resistance. *Enferm. Infecc. Microbiol. Clin.* 30 24–32. 10.1016/j.eimc.2011.07.017 22019131

[B17] Fredriksson-Ahomaa M. Cernela N. Hächler H. Stephan R. (2012). *Yersinia enterocolitica* strains associated with human infections in Switzerland 2001-2010. *Eur. J. Clin. Microbiol. Infect. Dis.* 31 1543–1550. 22071910 10.1007/s10096-011-1476-7

[B18] Guo B. C. Liu X. Hao Q. Yan L. Q. Xie M. Y. Xue X. Y. (2011). Monitoring and analysis of *Yersinia enterocolitica* in Ningxia area. *Mod. Med. Health* 27 2724–2726.

[B19] Hall M. Chattaway M. A. Reuter S. Savin C. Strauch E. Carniel E. (2015). Use of whole-genus genome sequence data to develop a multilocus sequence typing tool that accurately identifies *Yersinia* isolates to the species and subspecies levels. *J. Clin. Microbiol.* 53 35–42. 10.1128/JCM.02395-14 25339391 PMC4290955

[B20] Hu Y. Liu L. Zhang X. Feng Y. Zong Z. (2017). In vitro activity of neomycin, streptomycin, paromomycin and apramycin against carbapenem-resistant *Enterobacteriaceae* clinical strains. *Front. Microbiol.* 8:2275. 10.3389/fmicb.2017.02275 29250040 PMC5715380

[B21] Karlsson P. A. Tano E. Jernberg C. Hickman R. A. Guy L. Järhult J. D. (2021). Molecular characterization of multidrug-resistant *Yersinia enterocolitica* from foodborne outbreaks in Sweden. *Front. Microbiol.* 12:664665. 10.3389/fmicb.2021.664665 34054769 PMC8155512

[B22] Lawley T. D. Wilkins B. M. Frost L. S. (2004). “Bacterial conjugation in gram-negative bacteria,” in *Plasmid Biology*, eds Funnell B. Philips G. (Washington, DC: ASM Press), 203–226.

[B23] Li R. Yu C. Li Y. Lam T. W. Yiu S. M. Kristiansen K. (2009). SOAP2: an improved ultrafast tool for short read alignment. *Bioinformatics* 25 1966–1967. 10.1093/BIOINFORMATICS/BTP336 19497933

[B24] Li Y. (2017). *Study on Hazard Factors Confirmation of Streptomycin Dregs and Resistance Mechanisms Induced by the Factors in Bacteria*. Ph.D. dissertation. Beijing: Chinese Academy of Agricultural Sciences.

[B25] Liang J. R. Wang X. Xiao Y. C. Cui Z. G. Xia S. L. Hao Q. (2012). Prevalence of *Yersinia enterocolitica* in pigs slaughtered in Chinese abattoirs. *Appl. Environ. Microbiol.* 78 2949–2956. 10.1128/AEM.07893-11 22327599 PMC3318836

[B26] McDermott P. F. Tyson G. H. Kabera C. Chen Y. Li C. Folster J. P. (2016). Whole-genome sequencing for detecting antimicrobial resistance in nontyphoidal *Salmonella*. *Antimicrob. Agents Chemother.* 60 5515–5520. 10.1128/AAC.01030-16 27381390 PMC4997858

[B27] Monaghan T. F. Rahman S. N. Agudelo C. W. Wein A. J. Lazar J. M. Everaert K. (2021). Foundational statistical principles in medical research: sensitivity, specificity, positive predictive value, and negative predictive value. *Medicina* 57:503. 10.3390/medicina57050503 34065637 PMC8156826

[B28] Peng Z. X. Zou M. Y. Xu J. Guan W. Y. Li Y. Liu D. R. (2018). Antimicrobial susceptibility and drug-resistance genes of *Yersinia* spp. of retailed poultry in 4 provinces of China. *Chin. J. Prev. Med.* 52 358–363. 10.3760/cma.j.issn.0253-9624.2018.04.006 29614601

[B29] Public Health England (2014). *UK Standards for Microbiology Investigations B 30: Investigation of Faecal Specimens for Enteric Pathogens.* London: Public Health England.

[B30] Robab A. Pourya G. Vahid S. Hasan H. Vahideh T. Yousef M. M. (2020). Molecular mechanisms associated with quinolone resistance in *Enterobacteriaceae*: review and update. *Trans. R. Soc. Trop. Med. Hyg.* 114 770–781. 10.1093/trstmh/traa041 32609840

[B31] Schroeder M. Brooks B. D. Brooks A. E. (2017). The complex relationship between virulence and antibiotic resistance. *Genes* 8:39. 10.3390/GENES8010039 28106797 PMC5295033

[B32] Stock I. Heisig P. Wiedemann B. (1999). Expression of beta-lactamases in *Yersinia enterocolitica* strains of biovars 2, 4 and 5. *J. Med. Microbiol.* 48 1023–1027. 10.1099/00222615-48-11-1023 10535647

[B33] Su M. Satola S. W. Read T. D. (2019). Genome-based prediction of bacterial antibiotic resistance. *J. Clin. Microbiol.* 57:e01405-18. 10.1128/JCM.01405-18 30381421 PMC6425178

[B34] Sun W. K. Bi Z. W. Kou Z. Q. Hou P. B. Hu B. Liu J. L. (2013). Antimicrobial agent susceptibility of 163 strains of *Yersinia enterocolitic*. *Chin. J. Zoonoses* 29 339–342.

[B35] Toukdarian A. (2004). “Plasmid strategies for broad-host-range replication in gram-negative bacteria,” in *Plasmid Biology*, eds Funnell B. Phillips G. (Washington, DC: ASM Press), 259–270.

[B36] Valentin-Weigand P. Heesemann J. Dersch P. (2014). Unique virulence properties of *Yersinia enterocolitica* O:3–an emerging zoonotic pathogen using pigs as preferred reservoir host. *Int. J. Med. Microbiol.* 304 824–834. 10.1016/J.IJMM.2014.07.008 25172222

[B37] Van Boeckel T. P. Brower C. Gilbert M. Grenfell B. T. Levin S. A. Robinson T. P. (2015). Global trends in antimicrobial use in food animals. *Proc. Natl. Acad. Sci. U.S.A.* 112 5649–5654. 10.1073/PNAS.1503141112 25792457 PMC4426470

[B38] Verbikova V. Borilova G. Babak V. Moravkova M. (2018). Prevalence, characterization and antimicrobial susceptibility of *Yersinia enterocolitica* and other *Yersinia* species found in fruits and vegetables from the European Union. *Food Control* 85 161–167.

[B39] Villa L. García-Fernández A. Fortini D. Carattoli A. (2010). Replicon sequence typing of IncF plasmids carrying virulence and resistance determinants. *J. Antimicrob. Chemother.* 65 2518–2529. 20935300 10.1093/jac/dkq347

[B40] Vranakis I. Goniotakis I. Psaroulaki A. Sandalakis V. Tselentis Y. Gevaert K. (2014). Proteome studies of bacterial antibiotic resistance mechanisms. *J. Proteomics* 97 88–99. 10.1016/J.JPROT.2013.10.027 24184230

[B41] Wang X. Cui Z. Jin D. Tang L. Xia S. Wang H. (2009). Distribution of pathogenic *Yersinia enterocolitica* in China. *Eur. J. Clin. Microbiol. Infect. Dis.* 28 1237–1244. 10.1007/S10096-009-0773-X 19575249

[B42] Woolhouse M. Ward M. van Bunnik B. Farra J. (2015). Antimicrobial resistance in humans, livestock and the wider environment. *Philos. Trans. R. Soc. Lond. B Biol. Sci.* 370:20140083. 10.1098/RSTB.2014.0083 25918441 PMC4424433

[B43] Xiao Y. C. Wang X. Qiu H. Y. Jing H. Q. (2010). Study of biotyping for pathogenic *Y.enterocolitica* strains in China. *Chin. J. Zoonoses* 26 651–653.

[B44] Ye Q. H. Wu Q. P. Hu H. J. Zhang J. M. Huang H. X. (2015). Prevalence, antimicrobial resistance and genetic diversity of *Yersinia enterocolitica* isolated from retail frozen foods in China. *FEMS Microbiol. Lett.* 362:fnv197. 10.1093/FEMSLE/FNV197 26472688

[B45] Zhao S. Tyson G. H. Chen Y. S. Li C. Mukherjee S. Young S. (2015). Whole-genome sequencing analysis accurately predicts antimicrobial resistance phenotypes in *Campylobacter* spp. *Appl. Environ. Microbiol.* 82 459–466. 10.1128/AEM.02873-15 26519386 PMC4711122

[B46] Zhou Z. M. Alikhan N. F. Mohamed K. Fan Y. L. Achtman M. (2020). The EnteroBase user’s guide, with case studies on *Salmonella* transmissions, *Yersinia pestis* phylogeny, and *Escherichia* core genomic diversity. *Genome Res.* 30 138–152. 10.1101/GR.251678.119/-/DC1 31809257 PMC6961584

