# Supplementary material for: Whole-genome sequencing-based prediction and analysis of antimicrobial resistance in Yersinia enterocolitica from Ningxia, China
Source: Front Microbiol. 2022 Jul 22;13:936425. doi: 10.3389/fmicb.2022.936425 (PMC9356307; doi:10.3389/fmicb.2022.936425)
Supplement: Supplementary file 1 [file Data_Sheet_1.docx]

**Table S1.** The breakpoints of antibacterial agents

| Antibacterial agents | S (μg/mL) | I (μg/mL) | R (μg/mL) |
| --- | --- | --- | --- |
| Ampicillin (AMP) | ≦8 | 16 | ≧32 |
| Ampicillin/sulbactam (SAM) | ≦8/4 | 16/8 | ≧32/16 |
| Tetracycline (TET) | ≦4 | 8 | ≧16 |
| Cefazolin (CZO) | ≦2 | 4 | ≧8 |
| Cefuroxime (CXM) | ≦8 | 16 | ≧32 |
| Ceftazidime (CAZ) | ≦4 | 8 | ≧16 |
| Trimethoprim/sulfamethoxazole (SXT) | ≦2/38 | - | ≧4/76 |
| Polymyxin (POL) | - | ≦2 | ≧4 |
| Ciprofloxacin (CIP) | ≦0.25 | 0.5 | ≧1 |
| Gentamicin (GEN) | ≦4 | 8 | ≧16 |
| Chloramphenicol (CHL) | ≦8 | 16 | ≧32 |
| Imipenem (IPM) | ≦1 | 2 | ≧4 |
| Streptomycin (STR) | ≦32 | - | ≧64 |

R, resistant; I, intermediate resistant; S, susceptibility.

**Table S2.** List of WGS quality statistics

| Samplename | Total Num  (>500bp) | Total Length  (bp) | N50 Length  (bp) | N90 Length  (bp) | Sequence GC% | Clean Data Q30  (%) |
| --- | --- | --- | --- | --- | --- | --- |
| NX0738 | 145 | 4,530,111 | 61,450 | 16,003 | 46.95 | 90.85 |
| NX0740 | 141 | 4,533,813 | 62,544 | 17,729 | 46.97 | 90.89 |
| NX0741 | 131 | 4,527,800 | 62,118 | 17,868 | 46.92 | 87.95 |
| NX0742 | 119 | 4,591,669 | 67,295 | 20,470 | 46.93 | 92.4 |
| NX0743 | 108 | 4,541,947 | 76,330 | 22,156 | 46.88 | 91.46 |
| NX0744 | 130 | 4,528,241 | 62,551 | 18,276 | 46.91 | 89.34 |
| NX0745 | 117 | 4,536,515 | 64,093 | 21,384 | 46.89 | 91.66 |
| NX0746 | 115 | 4,547,932 | 71,025 | 20,445 | 46.92 | 91.18 |
| NX0747 | 109 | 4,530,806 | 72,235 | 22,156 | 46.89 | 91.13 |
| NX0748 | 104 | 4,539,437 | 74,357 | 25,025 | 46.88 | 88.71 |
| NX0749 | 106 | 4,540,830 | 76,330 | 24,095 | 46.88 | 90.7 |
| NX0750 | 109 | 4,546,561 | 72,091 | 22,156 | 46.92 | 89.03 |
| NX0751 | 103 | 4,538,064 | 74,357 | 24,095 | 46.87 | 93.07 |
| NX0752 | 108 | 4,548,953 | 72,091 | 23,949 | 46.92 | 93.39 |
| NX0753 | 108 | 4,536,608 | 71,914 | 24,095 | 46.88 | 93.28 |
| NX0754 | 112 | 4,523,224 | 73,194 | 20,360 | 46.9 | 93.04 |
| NX0755 | 126 | 4,533,405 | 69,421 | 18,276 | 46.9 | 93.51 |
| NX0756 | 113 | 4,527,466 | 71,005 | 21,279 | 46.88 | 92.81 |
| NX08020 | 120 | 4,530,608 | 66,691 | 19,283 | 46.9 | 93.11 |
| NX09001 | 128 | 4,541,445 | 63,557 | 18,264 | 46.94 | 93.07 |
| NX09002 | 116 | 4,547,996 | 70,951 | 21,276 | 46.92 | 93.3 |
| NX09003 | 108 | 4,548,761 | 73,194 | 24,095 | 46.92 | 93.85 |
| NX09004 | 114 | 4,546,685 | 72,225 | 21,276 | 46.92 | 93.56 |
| NX09005 | 105 | 4,544,453 | 72,225 | 24,095 | 46.91 | 92.99 |
| NX09006 | 108 | 4,550,082 | 73,194 | 24,095 | 46.91 | 92.43 |
| NX09007 | 27 | 4,645,756 | 297,328 | 117,924 | 47.17 | 93.07 |
| NX09008 | 49 | 4,798,156 | 191,525 | 52,644 | 47.17 | 92.66 |
| NX09009 | 79 | 4,897,604 | 196,740 | 38,341 | 47.21 | 92.71 |
| NX09010 | 112 | 4,838,563 | 108,386 | 29,126 | 47.32 | 92.93 |
| NX09011 | 81 | 4,900,005 | 196,740 | 36,216 | 47.22 | 93.1 |
| NX09013 | 19 | 4,662,935 | 636,181 | 150,981 | 47.05 | 93.22 |
| NX09014 | 38 | 4,763,912 | 309,965 | 99,305 | 47.35 | 93.1 |
| NX09017 | 47 | 4,714,918 | 169,370 | 67,703 | 47.11 | 93.36 |
| NX09018 | 18 | 4,760,897 | 559,382 | 202,771 | 47.13 | 93.51 |
| NX09028 | 95 | 4,451,793 | 77,902 | 24,075 | 46.96 | 93.04 |
| NX09029 | 105 | 4,518,098 | 77,384 | 23,976 | 46.92 | 92.96 |
| NX09030 | 125 | 4,539,161 | 63,557 | 17,871 | 46.95 | 93.21 |
| NX09036 | 22 | 4,600,648 | 579,191 | 150,881 | 47.09 | 93.44 |
| NX09037 | 55 | 4,936,558 | 215,896 | 61,263 | 47.11 | 93.37 |
| NX09038 | 22 | 5,017,938 | 337,439 | 120,823 | 47.16 | 92.85 |
| NX09039 | 30 | 4,927,868 | 556,322 | 151,010 | 47.08 | 91.21 |
| NX09040 | 42 | 4,679,754 | 243,508 | 88,829 | 47.17 | 93.01 |
| NX09041 | 35 | 4,757,094 | 185,604 | 84,480 | 47.22 | 93.08 |
| NX09042 | 28 | 4,730,955 | 404,338 | 129,558 | 47.18 | 92.93 |
| NX09043 | 29 | 4,705,861 | 299,683 | 128,142 | 47.21 | 92.36 |
| NX09044 | 28 | 4,699,624 | 320,020 | 127,853 | 47.21 | 93.19 |
| NX09045 | 19 | 4,704,771 | 320,013 | 113,935 | 47.21 | 92.12 |
| NX09047 | 20 | 4,646,455 | 699,164 | 323,346 | 47.11 | 93.16 |
| NX09048 | 12 | 4,647,915 | 714,441 | 384,659 | 47.02 | 93.42 |
| NX09057 | 24 | 4,648,016 | 385,287 | 162,820 | 47.09 | 93.24 |
| NX09058 | 28 | 5,013,128 | 433,478 | 150,981 | 47.05 | 92.38 |
| NX09059 | 24 | 4,689,542 | 554,761 | 154,005 | 47.16 | 93.72 |
| NX09060 | 22 | 4,625,811 | 632,023 | 168,752 | 47.11 | 93.35 |
| NX09061 | 27 | 4,627,017 | 308,623 | 147,475 | 47.13 | 94.09 |
| NX09062 | 32 | 4,792,917 | 452,575 | 137,398 | 47.16 | 93.31 |
| NX10041 | 93 | 4,624,097 | 121,068 | 28,144 | 47.1 | 93.11 |
| NX10043 | 124 | 4,533,476 | 63,915 | 18,305 | 46.89 | 93.16 |
| NX11036 | 56 | 4,665,688 | 185,202 | 58,185 | 47.15 | 93.46 |
| NX11058 | 147 | 4,520,490 | 60,005 | 16,006 | 46.95 | 93.46 |
| Samplename | Total Num  (>500bp) | Total Length  (bp) | N50 Length  (bp) | N90 Length  (bp) | Sequence GC% | Clean Data Q30  (%) |
| NX11070 | 138 | 4,482,855 | 60,043 | 17,775 | 46.98 | 93.21 |
| NX11072 | 167 | 4,544,479 | 50,820 | 14,983 | 47 | 93.31 |
| NX11073 | 101 | 4,464,776 | 74,347 | 25,048 | 46.91 | 88.41 |
| NX11074 | 101 | 4,532,216 | 73,544 | 25,367 | 46.88 | 94.05 |
| NX11075 | 102 | 4,520,902 | 72,235 | 25,367 | 46.88 | 87.59 |
| NX11076 | 101 | 4,531,537 | 73,369 | 25,048 | 46.88 | 88.91 |
| NX11077 | 104 | 4,529,458 | 72,224 | 25,048 | 46.87 | 90.72 |
| NX11078 | 103 | 4,532,674 | 73,369 | 25,048 | 46.88 | 88.52 |
| NX11079 | 101 | 4,528,888 | 72,235 | 25,367 | 46.88 | 87.92 |
| NX11080 | 105 | 4,528,264 | 73,194 | 25,367 | 46.88 | 88.25 |
| NX11081 | 99 | 4,529,760 | 73,194 | 25,367 | 46.87 | 88.07 |
| NX11082 | 111 | 4,466,665 | 70,951 | 24,095 | 46.92 | 88.22 |
| NX11083 | 104 | 4,465,820 | 72,225 | 25,048 | 46.92 | 88.7 |
| NX11084 | 127 | 4,457,582 | 66,064 | 18,276 | 46.95 | 88.23 |
| NX11085 | 101 | 4,523,253 | 71,115 | 25,367 | 46.88 | 87.87 |
| NX11086 | 101 | 4,433,996 | 72,225 | 25,052 | 46.96 | 87.71 |
| NX11087 | 150 | 4,507,830 | 61,453 | 15,012 | 46.99 | 88.33 |
| NX11088 | 110 | 4,531,531 | 72,235 | 24,125 | 46.91 | 88.16 |
| NX11089 | 103 | 4,530,139 | 71,914 | 24,095 | 46.87 | 88.23 |
| NX11090 | 110 | 4,535,021 | 71,914 | 22,156 | 46.88 | 88.31 |
| NX11091 | 119 | 4,461,369 | 63,557 | 21,276 | 46.93 | 87.99 |
| NX11092 | 110 | 4,529,711 | 72,236 | 21,788 | 46.88 | 94.81 |
| NX11093 | 120 | 4,465,660 | 63,374 | 20,433 | 46.93 | 89.09 |
| NX11094 | 101 | 4,456,354 | 72,236 | 25,048 | 46.92 | 90.89 |
| NX12009 | 37 | 5,009,313 | 257,207 | 128,429 | 46.94 | 88.52 |
| NX12010 | 14 | 4,771,729 | 702,514 | 387,326 | 47.14 | 88.64 |
| NX12011 | 8 | 4,599,118 | 685,749 | 387,326 | 47.12 | 90.73 |
| NX12012 | 31 | 4,594,702 | 248,968 | 150,693 | 47.01 | 90.68 |
| NX12013 | 16 | 4,399,024 | 301,125 | 165,652 | 46.99 | 90.96 |
| NX12014 | 39 | 4,956,085 | 318,573 | 111,183 | 47.31 | 88.33 |
| NX12015 | 41 | 4,938,948 | 261,112 | 100,662 | 47.3 | 87.94 |
| NX12016 | 36 | 4,853,584 | 497,447 | 80,419 | 47.24 | 88.26 |
| NX12017 | 45 | 4,826,359 | 428,584 | 98,614 | 47.14 | 88.38 |
| NX12018 | 76 | 4,877,659 | 229,902 | 76,557 | 47.32 | 90.34 |
| NX12019 | 35 | 4,724,089 | 279,736 | 71,147 | 47.04 | 88.23 |
| NX12020 | 39 | 4,935,919 | 318,573 | 111,183 | 47.29 | 88.58 |
| NX12023 | 37 | 4,881,604 | 257,231 | 100,200 | 46.87 | 90.99 |
| NX12024 | 23 | 4,618,085 | 363,336 | 150,890 | 47.13 | 87.89 |
| NX12025 | 42 | 4,939,117 | 261,112 | 100,521 | 47.3 | 88.34 |
| NX12036 | 23 | 4,722,216 | 396,180 | 193,243 | 47 | 90.64 |
| NX12056 | 105 | 4,536,090 | 73,194 | 25,048 | 46.88 | 88.33 |
| NX12057 | 106 | 4,538,843 | 73,194 | 25,048 | 46.88 | 87.91 |
| NX12058 | 109 | 4,532,396 | 72,235 | 25,048 | 46.91 | 88.29 |
| NX12059 | 101 | 4,531,431 | 72,091 | 25,048 | 46.87 | 88.43 |
| NX12060 | 107 | 4,532,456 | 73,217 | 25,048 | 46.91 | 88.35 |
| NX12061 | 108 | 4,534,431 | 71,914 | 25,071 | 46.87 | 88.89 |
| NX12062 | 107 | 4,532,654 | 73,193 | 25,071 | 46.91 | 88.71 |
| NX12063 | 106 | 4,534,731 | 73,194 | 24,095 | 46.88 | 87.04 |
| NX12064 | 31 | 4,741,927 | 546,309 | 151,097 | 47.12 | 87.64 |
| NX12065 | 32 | 4,735,587 | 416,586 | 151,361 | 47.17 | 88.66 |
| NX13049 | 25 | 4,692,688 | 323,698 | 117,841 | 47.23 | 87.93 |
| NX14046 | 47 | 4,735,947 | 392,307 | 100,527 | 47.16 | 87.9 |
| NX14047 | 44 | 4,733,684 | 461,118 | 100,527 | 47.15 | 88.24 |
| NX14050 | 46 | 4,735,409 | 461,252 | 100,527 | 47.16 | 88.29 |
| NX14056 | 52 | 4,734,730 | 312,353 | 77,998 | 47.16 | 88.1 |
| NX14109 | 143 | 4,786,719 | 72,792 | 19,275 | 46.73 | 87.94 |
| NX14111 | 125 | 4,748,156 | 72,303 | 20,116 | 46.73 | 87.9 |
| NX14112 | 142 | 4,840,701 | 68,777 | 18,177 | 46.76 | 87.93 |
| NX14113 | 28 | 4,816,381 | 421,420 | 120,890 | 46.87 | 88.71 |
| NX14114 | 22 | 4,640,802 | 551,502 | 127,879 | 47.24 | 88.13 |
| Samplename | Total Num  (>500bp) | Total Length  (bp) | N50 Length  (bp) | N90 Length  (bp) | Sequence GC% | Clean Data Q30  (%) |
| NX14115 | 152 | 4,819,194 | 73,052 | 18,078 | 46.76 | 86.73 |
| NX14116 | 161 | 4,798,568 | 66,149 | 17,975 | 46.74 | 88.24 |
| NX14117 | 157 | 4,865,033 | 70,980 | 18,177 | 46.75 | 90.18 |
| NX14118 | 144 | 4,815,335 | 72,345 | 19,153 | 46.75 | 87.85 |
| NX14121 | 21 | 4,679,473 | 608,565 | 154,155 | 47.25 | 93.73 |
| NX15095 | 31 | 4,693,011 | 535,668 | 150,929 | 47.32 | 94.03 |
| NX15096 | 10 | 4,829,820 | 677,544 | 396,876 | 47.17 | 94.63 |
| NX15097 | 9 | 4,830,200 | 677,546 | 396,876 | 47.17 | 88.48 |
| NX15098 | 13 | 4,831,037 | 588,889 | 268,397 | 47.17 | 88.05 |
| NX15099 | 26 | 4,907,132 | 517,192 | 112,688 | 46.8 | 88.29 |
| NX15100 | 7 | 4,790,027 | 677,539 | 396,876 | 47.15 | 87.47 |
| NX15106 | 16 | 4,619,743 | 665,116 | 171,387 | 46.99 | 88.52 |
| NX15108 | 19 | 4,755,704 | 519,281 | 385,290 | 46.88 | 88.63 |
| NX15132 | 24 | 4,764,092 | 359,316 | 150,542 | 47.15 | 88.43 |
| NX 16034 | 22 | 4,784,161 | 477,010 | 151,211 | 47.13 | 88.56 |
| NX16044 | 50 | 4,891,580 | 334,152 | 128,246 | 47.03 | 88.5 |
| NX16081 | 108 | 4,549,589 | 72,225 | 24,095 | 46.9 | 90.03 |
| NX16082 | 109 | 4,547,270 | 72,225 | 24,095 | 46.9 | 93.39 |
| NX16083 | 110 | 4,552,529 | 71,914 | 22,157 | 46.9 | 92.8 |
| NX16084 | 107 | 4,548,812 | 73,194 | 24,095 | 46.9 | 91.12 |
| NX16106 | 33 | 4,689,917 | 397,407 | 151,241 | 47.06 | 88.53 |
| NX17027 | 40 | 4,928,222 | 318,574 | 111,183 | 47.31 | 88.57 |
| NX17061 | 34 | 4,686,690 | 347,541 | 128,472 | 47.12 | 88.41 |
| NX17062 | 37 | 4,868,703 | 233,368 | 120,277 | 46.93 | 88.13 |
| NX17063 | 28 | 4,555,234 | 328,453 | 126,089 | 47.09 | 88.68 |
| NX17064 | 23 | 4,665,267 | 411,437 | 150,542 | 47.14 | 87.99 |
| NX17067 | 22 | 4,666,518 | 446,205 | 157,504 | 47.14 | 88.21 |
| NX17071 | 25 | 4,726,904 | 370,274 | 150,696 | 47.1 | 89.01 |
| NX17074 | 33 | 4,647,821 | 266,212 | 94,879 | 47.12 | 88.6 |
| NX18077 | 22 | 4,715,410 | 586,896 | 150,905 | 47.06 | 88.46 |
| NX18080 | 33 | 4,738,149 | 277,093 | 132,381 | 47.16 | 88.6 |
| NX18083 | 12 | 4,745,455 | 705,353 | 356,008 | 46.95 | 87.99 |
| NX18084 | 112 | 4,683,160 | 75,983 | 25,048 | 47.07 | 87.95 |
| NX18085 | 110 | 4,553,427 | 73,194 | 24,095 | 46.92 | 88.25 |
| NX18106 | 40 | 4,685,028 | 236,169 | 78,999 | 47.01 | 88.35 |
| NX18107 | 56 | 4,865,131 | 305,097 | 89,082 | 47.23 | 88.38 |
| NX18108 | 113 | 4,630,552 | 74,410 | 25,052 | 47 | 88.73 |
| NX18109 | 111 | 4,691,581 | 76,331 | 25,367 | 47.07 | 89.16 |
| NX18110 | 105 | 4,681,717 | 72,214 | 25,048 | 47.06 | 88.4 |
| NX18118 | 115 | 4,673,385 | 70,556 | 19,710 | 47.11 | 88.73 |
| NX18160 | 107 | 4,548,150 | 71,914 | 25,052 | 46.9 | 88.92 |
| NX18161 | 107 | 4,549,780 | 72,225 | 24,095 | 46.9 | 88.07 |
| NX18162 | 100 | 4,532,393 | 72,225 | 25,048 | 46.87 | 90.91 |
| NX18163 | 108 | 4,550,203 | 73,194 | 24,095 | 46.91 | 88.6 |
| NX18164 | 111 | 4,549,384 | 72,225 | 24,095 | 46.9 | 87.97 |
| NX18165 | 101 | 4,544,129 | 73,194 | 24,095 | 46.9 | 88.24 |
| NX18166 | 109 | 4,564,598 | 72,225 | 24,095 | 46.91 | 88.73 |
| NX18167 | 108 | 4,549,751 | 73,194 | 24,095 | 46.9 | 88.48 |
| NX18168 | 116 | 4,536,828 | 70,942 | 20,480 | 46.88 | 88.84 |
| NX18169 | 136 | 4,541,263 | 61,992 | 17,344 | 46.94 | 88.86 |
| NX18170 | 120 | 4,545,349 | 69,448 | 18,580 | 46.91 | 88.59 |
| NX18171 | 113 | 4,476,930 | 70,942 | 20,503 | 46.95 | 88.75 |
| NX18172 | 135 | 4,539,903 | 62,070 | 16,147 | 46.93 | 88.81 |
| NX18176 | 122 | 4,537,627 | 65,990 | 18,580 | 46.91 | 88.43 |
| NX19005 | 50 | 4,693,464 | 193,590 | 80,515 | 47.15 | 88.19 |
| NX19006 | 26 | 5,097,156 | 416,891 | 151,222 | 47.15 | 87.97 |
| NX19018 | 55 | 4,901,129 | 188,455 | 54,918 | 47.31 | 87.19 |
| NX19019 | 127 | 4,496,124 | 63,311 | 23,263 | 46.91 | 88.39 |
| NX19029 | 94 | 5,004,694 | 149,157 | 35,092 | 47.39 | 88.32 |
| NX19042 | 38 | 4,852,709 | 355,306 | 129,078 | 47.08 | 88.13 |
| Samplename | Total Num  (>500bp) | Total Length  (bp) | N50 Length  (bp) | N90 Length  (bp) | Sequence GC% | Clean Data Q30  (%) |
| NX19043 | 58 | 4,845,865 | 160,288 | 77,810 | 47.12 | 90.91 |
| NX19045 | 47 | 4,802,028 | 234,105 | 73,917 | 47.03 | 89.13 |
| NX19048 | 32 | 4,939,294 | 443,960 | 123,343 | 47.09 | 87.38 |
| NX19053 | 31 | 4,805,060 | 401,252 | 151,592 | 47.12 | 88.56 |
| NX19054 | 11 | 4,748,657 | 733,044 | 237,440 | 47.01 | 88.22 |
| NX19106 | 18 | 4,681,430 | 663,988 | 176,789 | 47.06 | 94.75 |
| NX19107 | 33 | 4,875,099 | 337,551 | 151,452 | 47.08 | 94.6 |
| NX19109 | 30 | 4,754,050 | 497,462 | 157,508 | 47.15 | 95.13 |
| NX19111 | 32 | 4,743,792 | 460,855 | 151,247 | 47.15 | 95.07 |
| NX19112 | 28 | 4,673,076 | 524,503 | 151,123 | 47.1 | 94.59 |

**TABLE S3.** List of 189 *Yersinia enterocolitica* isolates in the Ningxia Hui Autonomous Region

| **SRA** | **Samplename** | **Year** | **Origin** | **Location** | [**Coordinate**](C:/Users/%E6%9C%88%E5%84%BF/AppData/Local/youdao/dict/Application/8.9.6.0/resultui/html/index.html#/javascript:;) |
| --- | --- | --- | --- | --- | --- |
| SRR16693604 | NX0738 | 2007 | Pig | Yinchuan | 38.47 N 106.27 E |
| SRR16693603 | NX0740 | 2007 | Pig | Yinchuan | 38.47 N 106.27 E |
| SRR16693492 | NX0741 | 2007 | Pig | Yinchuan | 38.47 N 106.27 E |
| SRR16693404 | NX0742 | 2007 | Pig | Yinchuan | 38.47 N 106.27 E |
| SRR16693393 | NX0743 | 2007 | Pig | Yinchuan | 38.47 N 106.27 E |
| SRR16693382 | NX0744 | 2007 | Pig | Yinchuan | 38.47 N 106.27 E |
| SRR16693371 | NX0745 | 2007 | Pig | Yinchuan | 38.47 N 106.27 E |
| SRR16693360 | NX0746 | 2007 | Pig | Yinchuan | 38.47 N 106.27 E |
| SRR16693349 | NX0747 | 2007 | Pig | Yinchuan | 38.47 N 106.27 E |
| SRR16693338 | NX0748 | 2007 | Pig | Yinchuan | 38.47 N 106.27 E |
| SRR16693602 | NX0749 | 2007 | Pig | Yinchuan | 38.47 N 106.27 E |
| SRR16693591 | NX0750 | 2007 | Pig | Yinchuan | 38.47 N 106.27 E |
| SRR16693580 | NX0751 | 2007 | Pig | Yinchuan | 38.47 N 106.27 E |
| SRR16693569 | NX0752 | 2007 | Pig | Yinchuan | 38.47 N 106.27 E |
| SRR16693558 | NX0753 | 2007 | Pig | Yinchuan | 38.47 N 106.27 E |
| SRR16693547 | NX0754 | 2007 | Pig | Yinchuan | 38.47 N 106.27 E |
| SRR16693536 | NX0755 | 2007 | Pig | Yinchuan | 38.47 N 106.27 E |
| SRR16693525 | NX0756 | 2007 | Pig | Yinchuan | 38.47 N 106.27 E |
| SRR16693503 | NX08020 | 2008 | Pig | Yinchuan | 38.47 N 106.27 E |
| SRR16693491 | NX09001 | 2009 | Pig | Yinchuan | 38.10 N 106.34 E |
| SRR16693480 | NX09002 | 2009 | Pig | Yinchuan | 38.10 N 106.34 E |
| SRR16693469 | NX09003 | 2009 | Pig | Yinchuan | 38.10 N 106.34 E |
| SRR16693458 | NX09004 | 2009 | Pig | Yinchuan | 38.10 N 106.34 E |
| SRR16693447 | NX09005 | 2009 | Pig | Yinchuan | 38.10 N 106.34 E |
| SRR16693436 | NX09006 | 2009 | Pig | Yinchuan | 38.10 N 106.34 E |
| SRR16693425 | NX09007 | 2009 | Pig | Yinchuan | 38.10 N 106.34 E |
| SRR16693414 | NX09008 | 2009 | Pig | Yinchuan | 38.10 N 106.34 E |
| SRR16693406 | NX09009 | 2009 | Pig | Yinchuan | 38.10 N 106.34 E |
| SRR16693405 | NX09010 | 2009 | Pig | Yinchuan | 38.10 N 106.34 E |
| SRR16693403 | NX09011 | 2009 | Pig | Yinchuan | 38.10 N 106.34 E |
| SRR16693402 | NX09013 | 2009 | Pig | Zhongwei | 36.56 N 105.64 E |
| SRR16693401 | NX09014 | 2009 | Pig | Zhongwei | 36.56 N 105.64 E |
| SRR16693400 | NX09017 | 2009 | Pig | Zhongwei | 36.56 N 105.64 E |
| SRR16693399 | NX09018 | 2009 | Pig | Zhongwei | 36.56 N 105.64 E |
| SRR16693396 | NX09028 | 2009 | Pig | Zhongwei | 37.52 N 105.17 E |
| SRR16693395 | NX09029 | 2009 | Pig | Zhongwei | 37.52 N 105.17 E |
| SRR16693394 | NX09030 | 2009 | Pig | Zhongwei | 37.52 N 105.17 E |
| SRR16693390 | NX09036 | 2009 | Pig | Zhongwei | 36.56 N 105.64 E |
| SRR16693389 | NX09037 | 2009 | Pig | Zhongwei | 36.56 N 105.64 E |
| SRR16693388 | NX09038 | 2009 | Pig | Zhongwei | 36.56 N 105.64 E |
| SRR16693387 | NX09039 | 2009 | Pig | Zhongwei | 36.56 N 105.64 E |
| SRR16693386 | NX09040 | 2009 | Pig | Zhongwei | 36.56 N 105.64 E |
| SRR16693385 | NX09041 | 2009 | Pig | Zhongwei | 36.56 N 105.64 E |
| SRR16693384 | NX09042 | 2009 | Pig | Zhongwei | 36.56 N 105.64 E |
| SRR16693383 | NX09043 | 2009 | Pig | Zhongwei | 36.56 N 105.64 E |
| SRR16693381 | NX09044 | 2009 | Pig | Zhongwei | 36.56 N 105.64 E |
| SRR16693380 | NX09045 | 2009 | Pig | Zhongwei | 36.56 N 105.64 E |
| SRR16693379 | NX09047 | 2009 | Pig | Zhongwei | 36.56 N 105.64 E |
| SRR16693378 | NX09048 | 2009 | Pig | Zhongwei | 36.56 N 105.64 E |
| SRR16693377 | NX09057 | 2009 | Pig | Zhongwei | 36.56 N 105.64 E |
| SRR16693376 | NX09058 | 2009 | Pig | Zhongwei | 36.56 N 105.64 E |
| SRR16693375 | NX09059 | 2009 | Pig | Zhongwei | 36.56 N 105.64 E |
| SRR16693374 | NX09060 | 2009 | Pig | Zhongwei | 36.56 N 105.64 E |
| SRR16693373 | NX09061 | 2009 | Pig | Zhongwei | 36.56 N 105.64 E |
| SRR16693372 | NX09062 | 2009 | Pig | Zhongwei | 36.56 N 105.64 E |
| SRR16693366 | NX10041 | 2010 | Pig | Zhongwei | 37.52 N 105.17 E |
| SRR16693364 | NX10043 | 2010 | Pig | Zhongwei | 37.52 N 105.17 E |
| SRR16693361 | NX11036 | 2011 | Pig | Zhongwei | 36.56 N 105.64 E |
| SRR16693351 | NX11058 | 2011 | Pig | Zhongwei | 36.56 N 105.64 E |
| **SRA** | **Samplename** | **Year** | **Origin** | **Location** | [**Coordinate**](C:/Users/%E6%9C%88%E5%84%BF/AppData/Local/youdao/dict/Application/8.9.6.0/resultui/html/index.html#/javascript:;) |
| SRR16693350 | NX11070 | 2011 | Pig | Yinchuan | 38.47 N 106.27 E |
| SRR16693347 | NX11072 | 2011 | Pig | Yinchuan | 38.47 N 106.27 E |
| SRR16693346 | NX11073 | 2011 | Pig | Yinchuan | 38.47 N 106.27 E |
| SRR16693345 | NX11074 | 2011 | Pig | Yinchuan | 38.47 N 106.27 E |
| SRR16693344 | NX11075 | 2011 | Pig | Yinchuan | 38.47 N 106.27 E |
| SRR16693343 | NX11076 | 2011 | Pig | Yinchuan | 38.47 N 106.27 E |
| SRR16693342 | NX11077 | 2011 | Pig | Yinchuan | 38.47 N 106.27 E |
| SRR16693341 | NX11078 | 2011 | Pig | Yinchuan | 38.47 N 106.27 E |
| SRR16693340 | NX11079 | 2011 | Pig | Yinchuan | 38.47 N 106.27 E |
| SRR16693339 | NX11080 | 2011 | Pig | Yinchuan | 38.47 N 106.27 E |
| SRR16693337 | NX11081 | 2011 | Pig | Yinchuan | 38.47 N 106.27 E |
| SRR16693336 | NX11082 | 2011 | Pig | Yinchuan | 38.47 N 106.27 E |
| SRR16693335 | NX11083 | 2011 | Pig | Yinchuan | 38.47 N 106.27 E |
| SRR16693334 | NX11084 | 2011 | Pig | Yinchuan | 38.47 N 106.27 E |
| SRR16693333 | NX11085 | 2011 | Pig | Yinchuan | 38.47 N 106.27 E |
| SRR16693332 | NX11086 | 2011 | Pig | Yinchuan | 38.47 N 106.27 E |
| SRR16693331 | NX11087 | 2011 | Pig | Yinchuan | 38.47 N 106.27 E |
| SRR16693330 | NX11088 | 2011 | Pig | Yinchuan | 38.47 N 106.27 E |
| SRR16693329 | NX11089 | 2011 | Pig | Yinchuan | 38.47 N 106.27 E |
| SRR16693328 | NX11090 | 2011 | Pig | Yinchuan | 38.47 N 106.27 E |
| SRR16693601 | NX11091 | 2011 | Pig | Yinchuan | 38.47 N 106.27 E |
| SRR16693600 | NX11092 | 2011 | Pig | Yinchuan | 38.47 N 106.27 E |
| SRR16693599 | NX11093 | 2011 | Pig | Yinchuan | 38.47 N 106.27 E |
| SRR16693598 | NX11094 | 2011 | Pig | Yinchuan | 38.47 N 106.27 E |
| SRR16693597 | NX12009 | 2012 | Cattle | Zhongwei | 36.56 N 105.64 E |
| SRR16693596 | NX12010 | 2012 | Cattle | Zhongwei | 36.56 N 105.64 E |
| SRR16693595 | NX12011 | 2012 | Cattle | Zhongwei | 36.56 N 105.64 E |
| SRR16693594 | NX12012 | 2012 | Chicken | Zhongwei | 36.56 N 105.64 E |
| SRR16693593 | NX12013 | 2012 | Chicken | Zhongwei | 36.56 N 105.64 E |
| SRR16693592 | NX12014 | 2012 | Sheep | Zhongwei | 36.56 N 105.64 E |
| SRR16693590 | NX12015 | 2012 | Sheep | Zhongwei | 36.56 N 105.64 E |
| SRR16693589 | NX12016 | 2012 | Food | Yinchuan | 38.47 N 106.27 E |
| SRR16693588 | NX12017 | 2012 | Food | Yinchuan | 38.47 N 106.27 E |
| SRR16693587 | NX12018 | 2012 | Food | Yinchuan | 38.47 N 106.27 E |
| SRR16693586 | NX12019 | 2012 | Food | Yinchuan | 38.47 N 106.27 E |
| SRR16693585 | NX12020 | 2012 | Sheep | Zhongwei | 36.56 N 105.64 E |
| SRR16693582 | NX12023 | 2012 | Cattle | Zhongwei | 36.56 N 105.64 E |
| SRR16693581 | NX12024 | 2012 | Cattle | Zhongwei | 36.56 N 105.64 E |
| SRR16693579 | NX12025 | 2012 | Sheep | Zhongwei | 36.56 N 105.64 E |
| SRR16693578 | NX12036 | 2012 | Pig | Zhongwei | 36.56 N 105.64 E |
| SRR16693577 | NX12056 | 2012 | Pig | Yinchuan | 38.47 N 106.27 E |
| SRR16693576 | NX12057 | 2012 | Pig | Yinchuan | 38.47 N 106.27 E |
| SRR16693575 | NX12058 | 2012 | Pig | Yinchuan | 38.47 N 106.27 E |
| SRR16693574 | NX12059 | 2012 | Pig | Yinchuan | 38.47 N 106.27 E |
| SRR16693573 | NX12060 | 2012 | Pig | Yinchuan | 38.47 N 106.27 E |
| SRR16693572 | NX12061 | 2012 | Pig | Yinchuan | 38.47 N 106.27 E |
| SRR16693571 | NX12062 | 2012 | Pig | Yinchuan | 38.47 N 106.27 E |
| SRR16693570 | NX12063 | 2012 | Pig | Yinchuan | 38.47 N 106.27 E |
| SRR16693568 | NX12064 | 2012 | Food | Yinchuan | 38.47 N 106.27 E |
| SRR16693567 | NX12065 | 2012 | Food | Yinchuan | 38.47 N 106.27 E |
| SRR16693566 | NX13049 | 2013 | Food | Yinchuan | 38.47 N 106.27 E |
| SRR16693564 | NX14046 | 2014 | Rat | Zhongwei | 36.56 N 105.64 E |
| SRR16693563 | NX14047 | 2014 | Rat | Zhongwei | 36.56 N 105.64 E |
| SRR16693560 | NX14050 | 2014 | Rat | Zhongwei | 36.56 N 105.64 E |
| SRR16693553 | NX14056 | 2014 | Rat | Zhongwei | 36.56 N 105.64 E |
| SRR16693545 | NX14109 | 2014 | Sheep | Zhongwei | 36.56 N 105.64 E |
| SRR16693544 | NX14111 | 2014 | Sheep | Zhongwei | 36.56 N 105.64 E |
| SRR16693543 | NX14112 | 2014 | Sheep | Zhongwei | 36.56 N 105.64 E |
| SRR16693542 | NX14113 | 2014 | Pig | Zhongwei | 36.56 N 105.64 E |
| SRR16693541 | NX14114 | 2014 | Pig | Zhongwei | 36.56 N 105.64 E |
| **SRA** | **Samplename** | **Year** | **Origin** | **Location** | [**Coordinate**](C:/Users/%E6%9C%88%E5%84%BF/AppData/Local/youdao/dict/Application/8.9.6.0/resultui/html/index.html#/javascript:;) |
| SRR16693540 | NX14115 | 2014 | Sheep | Zhongwei | 36.56 N 105.64 E |
| SRR16693539 | NX14116 | 2014 | Sheep | Zhongwei | 36.56 N 105.64 E |
| SRR16693538 | NX14117 | 2014 | Sheep | Zhongwei | 36.56 N 105.64 E |
| SRR16693537 | NX14118 | 2014 | Sheep | Zhongwei | 36.56 N 105.64 E |
| SRR16693533 | NX14121 | 2014 | Pig | Zhongwei | 36.56 N 105.64 E |
| SRR16693532 | NX15095 | 2015 | Sheep | Zhongwei | 36.56 N 105.64 E |
| SRR16693531 | NX15096 | 2015 | Sheep | Zhongwei | 36.56 N 105.64 E |
| SRR16693530 | NX15097 | 2015 | Sheep | Zhongwei | 36.56 N 105.64 E |
| SRR16693529 | NX15098 | 2015 | Sheep | Zhongwei | 36.56 N 105.64 E |
| SRR16693528 | NX15099 | 2015 | Sheep | Zhongwei | 36.56 N 105.64 E |
| SRR16693527 | NX15100 | 2015 | Sheep | Zhongwei | 36.56 N 105.64 E |
| SRR16693523 | NX15106 | 2015 | Sheep | Zhongwei | 36.56 N 105.64 E |
| SRR16693521 | NX15108 | 2015 | Rat | Zhongwei | 36.56 N 105.64 E |
| SRR16693519 | NX15132 | 2015 | Pig | Zhongwei | 36.56 N 105.64 E |
| SRR16693517 | NX 16034 | 2016 | Food | Yinchuan | 38.47 N 106.27 E |
| SRR16693516 | NX16044 | 2016 | Cattle | Zhongwei | 36.56 N 105.64 E |
| SRR16693515 | NX16081 | 2016 | Pig | Zhongwei | 36.56 N 105.64 E |
| SRR16693513 | NX16082 | 2016 | Pig | Zhongwei | 36.56 N 105.64 E |
| SRR16693512 | NX16083 | 2016 | Pig | Zhongwei | 36.56 N 105.64 E |
| SRR16693511 | NX16084 | 2016 | Pig | Zhongwei | 36.56 N 105.64 E |
| SRR16693509 | NX16106 | 2016 | Food | Yinchuan | 38.47 N 106.27 E |
| SRR16693505 | NX17027 | 2017 | Chicken | Zhongwei | 36.56 N 105.64 E |
| SRR16693504 | NX17061 | 2017 | Pig | Zhongwei | 36.56 N 105.64 E |
| SRR16693502 | NX17062 | 2017 | Pig | Zhongwei | 36.56 N 105.64 E |
| SRR16693501 | NX17063 | 2017 | Pig | Zhongwei | 36.56 N 105.64 E |
| SRR16693500 | NX17064 | 2017 | Pig | Zhongwei | 36.56 N 105.64 E |
| SRR16693497 | NX17067 | 2017 | Pig | Zhongwei | 36.56 N 105.64 E |
| SRR16693493 | NX17071 | 2017 | Pig | Zhongwei | 36.56 N 105.64 E |
| SRR16693488 | NX17074 | 2017 | Pig | Zhongwei | 36.56 N 105.64 E |
| SRR16693485 | NX18077 | 2018 | Food | Yinchuan | 38.47 N 106.27 E |
| SRR16693482 | NX18080 | 2018 | Food | Yinchuan | 38.47 N 106.27 E |
| SRR16693479 | NX18083 | 2018 | Human | Wuzhong | 38.02 N 106.08 E |
| SRR16693478 | NX18084 | 2018 | Human | Shizuishan | 39.02 N 106.37 E |
| SRR16693477 | NX18085 | 2018 | Human | Shizuishan | 39.02 N 106.37 E |
| SRR16693476 | NX18106 | 2018 | Human | Guyuan | 36.00 N 106.29 E |
| SRR16693475 | NX18107 | 2018 | Human | Guyuan | 36.00 N 106.29 E |
| SRR16693474 | NX18108 | 2018 | Human | Zhongwei | 37.52 N 105.17 E |
| SRR16693473 | NX18109 | 2018 | Human | Shizuishan | 39.02 N 106.37 E |
| SRR16693472 | NX18110 | 2018 | Human | Shizuishan | 39.02 N 106.37 E |
| SRR16693471 | NX18118 | 2018 | Human | Shizuishan | 39.02 N 106.37 E |
| SRR16693463 | NX18160 | 2018 | Pig | Zhongwei | 36.56 N 105.64 E |
| SRR16693462 | NX18161 | 2018 | Pig | Zhongwei | 36.56 N 105.64 E |
| SRR16693461 | NX18162 | 2018 | Pig | Zhongwei | 36.56 N 105.64 E |
| SRR16693460 | NX18163 | 2018 | Pig | Zhongwei | 36.56 N 105.64 E |
| SRR16693459 | NX18164 | 2018 | Pig | Zhongwei | 36.56 N 105.64 E |
| SRR16693457 | NX18165 | 2018 | Pig | Zhongwei | 36.56 N 105.64 E |
| SRR16693456 | NX18166 | 2018 | Pig | Zhongwei | 36.56 N 105.64 E |
| SRR16693455 | NX18167 | 2018 | Pig | Zhongwei | 36.56 N 105.64 E |
| SRR16693454 | NX18168 | 2018 | Pig | Zhongwei | 36.56 N 105.64 E |
| SRR16693453 | NX18169 | 2018 | Pig | Zhongwei | 36.56 N 105.64 E |
| SRR16693452 | NX18170 | 2018 | Pig | Zhongwei | 36.56 N 105.64 E |
| SRR16693451 | NX18171 | 2018 | Pig | Zhongwei | 36.56 N 105.64 E |
| SRR16693450 | NX18172 | 2018 | Pig | Zhongwei | 36.56 N 105.64 E |
| SRR16693445 | NX18176 | 2018 | Human | Guyuan | 35.85 N 106.64 E |
| SRR16693442 | NX19005 | 2019 | Food | Yinchuan | 38.47 N 106.27 E |
| SRR16693441 | NX19006 | 2019 | Food | Yinchuan | 38.47 N 106.27 E |
| SRR16693439 | NX19018 | 2019 | Food | Shizuishan | 39.02 N 106.37 E |
| SRR16693438 | NX19019 | 2019 | Human | Yinchuan | 38.47 N 106.27 E |
| SRR16693434 | NX19029 | 2019 | Food | Yinchuan | 38.47 N 106.27 E |
| SRR16693433 | NX19042 | 2019 | Food | Yinchuan | 38.47 N 106.27 E |
| **SRA** | **Samplename** | **Year** | **Origin** | **Location** | [**Coordinate**](C:/Users/%E6%9C%88%E5%84%BF/AppData/Local/youdao/dict/Application/8.9.6.0/resultui/html/index.html#/javascript:;) |
| SRR16693432 | NX19043 | 2019 | Food | Yinchuan | 38.47 N 106.27 E |
| SRR16693430 | NX19045 | 2019 | Food | Yinchuan | 38.47 N 106.27 E |
| SRR16693427 | NX19048 | 2019 | Food | Yinchuan | 38.47 N 106.27 E |
| SRR16693421 | NX19053 | 2019 | Food | Yinchuan | 38.47 N 106.27 E |
| SRR16693420 | NX19054 | 2019 | Food | Yinchuan | 38.47 N 106.27 E |
| SRR16693413 | NX19106 | 2019 | Human | Zhongwei | 36.56 N 105.64 E |
| SRR16693412 | NX19107 | 2019 | Human | Zhongwei | 36.56 N 105.64 E |
| SRR16693410 | NX19109 | 2019 | Food | Yinchuan | 38.47 N 106.27 E |
| —— | NX19111 | 2019 | Food | Yinchuan | 38.47 N 106.27 E |
| SRR16693407 | NX19112 | 2019 | Food | Yinchuan | 38.47 N 106.27 E |

**TABLE S4.** Origin, serotype, STs, and CTs of 189 strains of *Y. enterocolitica*

| **ST** | **CT** | **Samplename** | **Origin** | **Biotype** | **Serotype** |
| --- | --- | --- | --- | --- | --- |
| 429 | 4609 | NX18160 | Pig | 4 | O: 3 |
|  |  | NX18161 | Pig | 4 | O: 3 |
|  |  | NX18163 | Pig | 4 | O: 3 |
|  |  | NX18164 | Pig | 4 | O: 3 |
|  |  | NX18165 | Pig | 4 | O: 3 |
|  |  | NX18167 | Pig | 4 | O: 3 |
|  |  | NX18168 | Pig | 4 | O: 3 |
|  |  | NX18169 | Pig | 4 | O: 3 |
|  |  | NX18171 | Pig | 4 | O: 3 |
|  |  | NX18166 | Pig | 4 | O: 3 |
|  | 4506 | NX09001 | Pig | 4 | O: 3 |
|  |  | NX09002 | Pig | 4 | O: 3 |
|  |  | NX09003 | Pig | 4 | O: 3 |
|  |  | NX09004 | Pig | 4 | O: 3 |
|  |  | NX09005 | Pig | 4 | O: 3 |
|  |  | NX09006 | Pig | 4 | O: 3 |
|  | 4493 | NX0740 | Pig | 4 | O: 3 |
|  |  | NX0742 | Pig | 4 | O: 3 |
|  |  | NX0746 | Pig | 4 | O: 3 |
|  |  | NX0750 | Pig | 4 | O: 3 |
|  |  | NX0752 | Pig | 3 | O: 3 |
|  | 4536 | NX11073 | Pig | 4 | O: 3 |
|  |  | NX11077 | Pig | 4 | O: 3 |
|  |  | NX11081 | Pig | 4 | O: 3 |
|  |  | NX11083 | Pig | 4 | O: 3 |
|  |  | NX11091 | Pig | 4 | O: 3 |
|  | 4534 | NX11072 | Pig | 4 | O: 3 |
|  |  | NX11074 | Pig | 4 | O: 3 |
|  |  | NX11078 | Pig | 4 | O: 3 |
|  |  | NX11082 | Pig | 3 | O: 3 |
|  | 4561 | NX12058 | Pig | 4 | O: 3 |
|  |  | NX12060 | Pig | 4 | O: 3 |
|  |  | NX12061 | Pig | 4 | O: 3 |
|  |  | NX12062 | Pig | 4 | O: 3 |
|  | 4589 | NX16081 | Pig | 4 | O: 3 |
|  |  | NX16082 | Pig | 4 | O: 3 |
|  |  | NX16083 | Pig | 4 | O: 3 |
|  |  | NX16084 | Pig | 4 | O: 3 |
|  | 4497 | NX0745 | Pig | 4 | O: 3 |
|  |  | NX0749 | Pig | 4 | O: 3 |
|  |  | NX0751 | Pig | 4 | O: 3 |
|  |  | NX0747 | Pig | 4 | O: 3 |
|  | 4537 | NX11075 | Pig | 4 | O: 3 |
|  |  | NX11080 | Pig | 4 | O: 3 |
|  |  | NX11085 | Pig | 4 | O: 3 |
|  | 4543 | NX11089 | Pig | 3 | O: 3 |
|  |  | NX11090 | Pig | 4 | O: 3 |
|  |  | NX11093 | Pig | 4 | O: 3 |
|  | 4502 | NX0753 | Pig | 4 | O: 3 |
|  |  | NX0756 | Pig | 4 | O: 3 |
|  | 4539 | NX11079 | Pig | 4 | O: 3 |
|  |  | NX11087 | Pig | 4 | O: 3 |
|  | 4544 | NX11092 | Pig | 4 | O: 3 |
|  |  | NX11094 | Pig | 4 | O: 3 |
|  | 4603 | NX18084 | Human | 4 | O: 3 |
|  |  | NX18109 | Human | 4 | O: 3 |
|  | 4560 | NX12059 | Pig | 4 | O: 3 |
|  |  | NX12057 | Pig | 4 | O: 3 |
|  | 4492 | NX0738 | Pig | 4 | O: 3 |
|  | 4494 | NX0741 | Pig | 4 | O: 3 |
| **ST** | **CT** | **Samplename** | **Origin** | **Biotype** | **Serotype** |
|  | 4495 | NX0743 | Pig | 4 | O: 3 |
|  | 4496 | NX0744 | Pig | 4 | O: 3 |
|  | 4498 | NX0748 | Pig | 4 | O: 3 |
|  | 4503 | NX0754 | Pig | 4 | O: 3 |
|  | 4505 | NX08020 | Pig | 4 | O: 3 |
|  | 4516 | NX09030 | Pig | 4 | O: 3 |
|  | 4533 | NX11070 | Pig | 4 | O: 3 |
|  | 4538 | NX11076 | Pig | 4 | O: 3 |
|  | 4540 | NX11084 | Pig | 4 | O: 3 |
|  | 4541 | NX11086 | Pig | 4 | O: 3 |
|  | 4542 | NX11088 | Pig | 4 | O: 3 |
|  | 4559 | NX12056 | Pig | 3 | O: 3 |
|  | 4562 | NX12063 | Pig | 4 | O: 3 |
|  | 4604 | NX18085 | Human | 4 | O: 3 |
|  | 4607 | NX18108 | Human | 4 | O: 3 |
|  | 4608 | NX18110 | Human | 4 | O: 3 |
|  | 4610 | NX18118 | Human | 4 | O: 3 |
|  | 4611 | NX18162 | Pig | 4 | O: 3 |
|  | 4614 | NX18170 | Pig | 4 | O: 3 |
|  | 4615 | NX18172 | Pig | 4 | O: 3 |
|  | 4616 | NX18176 | Human | 4 | O: 3 |
|  | 4746 | NX10043 | Pig | 4 | O: 3 |
| 3 | 4509 | NX09009 | Pig | 1A | O: 5 |
|  |  | NX09010 | Pig | 1A | O: 5 |
|  |  | NX09011 | Pig | 1A | O: 5 |
|  | 4750 | NX12017 | Food | 1A | O: 5 |
|  | 4579 | NX15095 | Sheep | 1A | O: 5 |
|  | 4594 | NX17062 | Pig | 1A | O: 5 |
|  | 4598 | NX17071 | Pig | 1A | O: 5 |
|  | 4618 | NX19018 | Food | 1A | O: 5 |
|  | 4759 | NX19029 | Food | 1A | O: 5 |
| 13 | 4570 | NX14109 | Sheep | 5 | O: 3 |
|  |  | NX14111 | Sheep | 5 | O: 3 |
|  | 4576 | NX14117 | Sheep | 5 | O: 3 |
|  |  | NX14118 | Sheep | 5 | O: 3 |
|  | 4571 | NX14112 | Sheep | 5 | O: 3 |
|  | 4752 | NX14115 | Sheep | 5 | O: 3 |
|  | 4575 | NX14116 | Sheep | 5 | O: 3 |
| 278 | 4527 | NX09047 | Pig | 1A | O: 5 |
|  | 4585 | NX15108 | Rat | 1A | O: 5 |
|  | 4754 | NX16034 | Food | 1A | O: 5 |
|  | 4755 | NX17061 | Pig | 1A | O: 5 |
|  | 4599 | NX17074 | Pig | 1A | O: 5 |
|  | 4758 | NX19006 | Food | 1A | Nag |
| 178 | 4630 | NX19109 | Food | 1A | O: 8 |
|  |  | NX19111 | Food | 1A | O: 5 |
|  | 4744 | NX09062 | Pig | 1A | O: 8 |
|  | 4551 | NX12016 | Food | 1A | O: 8 |
|  | 4591 | NX16106 | Food | 1A | O: 8 |
| 637 | 4550 | NX12014 | Sheep | 1A | O: 8 |
|  |  | NX12015 | Sheep | 1A | O: 8 |
|  |  | NX12020 | Sheep | 1A | O: 8 |
|  |  | NX12025 | Sheep | 1A | O: 8 |
|  | 4592 | NX17027 | Chicken | 1A | O: 8 |
| 640 | 4568 | NX14047 | Rat | 1A | O: 5 |
|  |  | NX14050 | Rat | 1A | O: 5 |
|  | 4569 | NX14046 | Rat | 1A | O: 5 |
|  |  | NX14056 | Rat | 1A | O: 5 |
| 643 | 4580 | NX15096 | Sheep | 1A | O: 8 |
|  |  | NX15098 | Sheep | 1A | O: 8 |
|  | 4581 | NX15097 | Sheep | 1A | O: 8 |
| **ST** | **CT** | **Samplename** | **Origin** | **Biotype** | **Serotype** |
|  |  | NX15100 | Sheep | 1A | O: 8 |
| 216 | 4596 | NX15132 | Pig | 1A | O: 5 |
|  |  | NX17064 | Pig | 1A | O: 5 |
|  |  | NX17067 | Pig | 1A | O: 5 |
| 541 | 4548 | NX12012 | Chicken | 1A | O: 5 |
|  |  | NX12013 | Chicken | 1A | O: 5 |
|  | 4756 | NX18106 | Human | 1A | O: 1,2,5 |
| 631 | 4739 | NX09043 | Pig | 1A | O: 8 |
|  |  | NX09044 | Pig | 1A | O: 8 |
|  |  | NX09045 | Pig | 1A | O: 8 |
| 145 | 4515 | NX09028 | Pig | 4 | O: 3 |
|  |  | NX09029 | Pig | 4 | O: 3 |
| 157 | 4751 | NX12018 | Food | 1A | O: 9 |
|  | 4606 | NX18107 | Human | 1A | O: 9 |
| 162 | 4521 | NX09040 | Pig | 1A | O: 5 |
|  | 4747 | NX11036 | Pig | 1A | O: 5 |
| 166 | 4738 | NX09038 | Pig | 1A | O: 5 |
|  | 4595 | NX17063 | Pig | 1A | O: 5 |
| 389 | 4622 | NX19042 | Food | 2 | O: 9 |
|  |  | NX19043 | Food | 2 | O: 9 |
| 26 | 4625 | NX19053 | Food | 2 | O: 9 |
| 563 | 4600 | NX18077 | Food | 1A | O: 53 |
|  | 4760 | NX19045 | Food | 1A | O: 5 |
| 600 | 4733 | NX09013 | Pig | 1A | O: 5 |
|  | 4745 | NX09058 | Pig | 1A | O: 5 |
| 625 | 4504 | NX0755 | Pig | 4 | O: 3 |
|  | 4532 | NX11058 | Pig | 4 | O: 3 |
| 18 | 4620 | NX19019 | Human | 4 | Nag |
| 633 | 4530 | NX09059 | Pig | 1A | O: 5 |
|  | 4742 | NX09061 | Pig | 1A | O: 5 |
| 634 | 4741 | NX09060 | Pig | 1A | O: 5 |
|  | 4582 | NX15099 | Sheep | 1A | O:5,8,9 |
| 635 | 4749 | NX12009 | Cattle | 1A | O: 5 |
|  | 4556 | NX12023 | Cattle | 1A | O: 5 |
| 636 | 4546 | NX12010 | Cattle | 1A | O: 5 |
|  |  | NX12011 | Cattle | 1A | O: 5 |
| 642 | 4572 | NX14114 | Pig | 1A | O: 5 |
|  | 4578 | NX14121 | Pig | 1A | O: 5 |
| 5 | 4564 | NX12065 | Food | 1A | O: 8 |
| 6 | 4511 | NX09014 | Pig | 1A | O: 5 |
| 8 | 4762 | NX19048 | Food | 1A | O: 5 |
| 19 | 4734 | NX09017 | Pig | 1A | O: 5 |
| 219 | 4523 | NX09042 | Pig | 1A | O: 5 |
| 209 | 4531 | NX10041 | Pig | 1A | O: 5 |
| 293 | 4557 | NX12024 | Cattle | 1A | O: 5 |
| 306 | 4601 | NX18080 | Food | 1A | Nag |
| 311 | 4554 | NX12019 | Food | 1A | O: 5 |
| 360 | 4558 | NX12036 | Pig | 1A | O: 5 |
| 404 | 4735 | NX09018 | Pig | 1A | O: 5 |
| 415 | 4737 | NX09039 | Pig | 1A | O: 5 |
| 480 | 4584 | NX15106 | Sheep | 1A | O: 8 |
| 493 | 4602 | NX18083 | Human | 1A | Nag |
| 536 | 4588 | NX16044 | Cattle | 1A | O: 5 |
| 556 | 4626 | NX19054 | Food | 1A | O: 8 |
| 626 | 4507 | NX09007 | Pig | 1A | O: 5 |
| 627 | 4508 | NX09008 | Pig | 1A | O: 5 |
| 628 | 4517 | NX09036 | Pig | 1A | O: 5 |
| 629 | 4736 | NX09037 | Pig | 1A | O: 5 |
| 630 | 4740 | NX09041 | Pig | 1A | O: 5 |
| 632 | 4748 | NX09048 | Pig | 1A | O: 5 |
| 638 | 4563 | NX12064 | Food | 1A | O: 8 |
| **ST** | **CT** | **Samplename** | **Origin** | **Biotype** | **Serotype** |
| 639 | 4565 | NX13049 | Food | 1A | O: 5 |
| 641 | 4753 | NX14113 | Pig | 1A | O: 5 |
| 644 | 4619 | NX19005 | Food | 1A | Nag |
| 645 | 4628 | NX19106 | Human | 1A | Nag |
| 646 | 4597 | NX19112 | Food | 1A | O: 5 |
| 655 | 4743 | NX09057 | Pig | 1A | O: 8 |
| 656 | 4761 | NX19107 | Human | 1A | O: 8 |

STs, sequences types; CTs, cgMLST types; Nag, nonagglutinative.

**TABLE S5.** Phenotypic antimicrobial resistance pattern of 189 *Y. enterocolitica* strains

| **Isolate** | **AMP** | **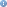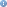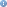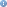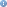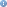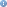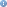SAM** | **TET** | **CZO** | **CXM** | **CAZ** | **SXT** | **POL** | **CIP** | **GEN** | **STR** | **CHL** | **IPM** |
| --- | --- | --- | --- | --- | --- | --- | --- | --- | --- | --- | --- | --- | --- |
| NX0738 | 16 (I) | 8/4 (S) | 64 (R) | >64 (R) | 1/19 (S) | 0.5 (I) | 0.5 (I) | <0.12 (S) | 4 (S) | 0.5 (S) | >128 (R) | 2 (S) | 0.5 (S) |
| NX0740 | 4 (S) | 4/2 (S) | 1 (S) | 2 (S) | <0.06/1.2 (S) | 0.5 (I) | 0.5 (I) | 1 (S) | 2 (S) | <0.25 (S) | >128 (R) | 2 (S) | 0.5 (S) |
| NX0741 | 16 (I) | 4/2 (S) | 1 (S) | >64 (R) | 0.12/2.4 (S) | 1 (I) | <0.12 (S) | <0.12 (S) | 4 (S) | 0.5 (S) | 8 (S) | 4 (S) | 0.5 (S) |
| NX0742 | 8 (S) | 4/2 (S) | <0.5 (S) | 32 (R) | <0.06/1.2 (S) | 0.5 (I) | 0.5 (I) | <0.12 (S) | 4 (S) | <0.25 (S) | >128 (R) | 2 (S) | 0.5 (S) |
| NX0743 | 16 (I) | 4/2 (S) | 1 (S) | 64 (R) | <0.06/1.2 (S) | 0.5 (I) | <0.12 (S) | <0.12 (S) | 2 (S) | 0.5 (S) | 8 (S) | 4 (S) | 0.5 (S) |
| NX0744 | 16 (I) | 4/2 (S) | 1 (S) | >64 (R) | <0.06/1.2 (S) | 0.5 (I) | <0.12 (S) | <0.12 (S) | 4 (S) | 0.5 (S) | 4 (S) | 4 (S) | 0.5 (S) |
| NX0745 | 16 (I) | 2/1 (S) | 1 (S) | 64 (R) | <0.06/1.2 (S) | 0.5 (I) | <0.12 (S) | <0.12 (S) | 2 (S) | 0.5 (S) | 8 (S) | 2 (S) | 0.5 (S) |
| NX0746 | 32 (R) | 16/8 (I) | 64 (R) | >64 (R) | 2/38 (S) | 1 (I) | 2 (R) | 0.25 (S) | 8 (S) | 0.5 (S) | >128 (R) | 4 (S) | 0.5 (S) |
| NX0747 | 16 (I) | 4/2 (S) | 1 (S) | 64 (R) | 0.12/2.4 (S) | 0.5 (I) | <0.12 (S) | <0.12 (S) | 2 (S) | 1 (S) | 4 (S) | 4 (S) | 0.5 (S) |
| NX0748 | 8 (S) | 2/1 (S) | <0.5 (S) | 32 (R) | <0.06/1.2 (S) | 0.5 (I) | <0.12 (S) | <0.12 (S) | 2 (S) | <0.25 (S) | 8 (S) | 2 (S) | 0.5 (S) |
| NX0749 | 8 (S) | 8/4 (S) | <0.5 (S) | 32 (R) | 0.12/2.4 (S) | 0.5 (I) | <0.12 (S) | <0.12 (S) | 2 (S) | <0.25 (S) | 8 (S) | 2 (S) | 0.5 (S) |
| NX0750 | 16 (I) | 8/4 (S) | <0.5 (S) | <0.5 (S) | 2/38 (S) | 0.5 (I) | 2 (R) | <0.12 (S) | 4 (S) | 0.5 (S) | >128 (R) | 4 (S) | 0.5 (S) |
| NX0751 | 16 (I) | 4/2 (S) | 1 (S) | <0.5 (S) | 0.12/2.4 (S) | 0.5 (I) | <0.12 (S) | <0.12 (S) | 2 (S) | 0.5 (S) | 8 (S) | 4 (S) | 0.5 (S) |
| NX0752 | 16 (I) | 4/2 (S) | 32 (R) | 64 (R) | 0.12/2.4 (S) | 0.5 (I) | <0.12 (S) | <0.12 (S) | 2 (S) | <0.25 (S) | >128 (R) | 4 (S) | 0.5 (S) |
| NX0753 | 16 (I) | 4/2 (S) | <0.5 (S) | >64 (R) | 0.12/2.4 (S) | 0.5 (I) | <0.12 (S) | <0.12 (S) | 2 (S) | 0.5 (S) | 8 (S) | 2 (S) | 0.5 (S) |
| NX0754 | 16 (I) | 4/2 (S) | 1 (S) | 64 (R) | <0.06/1.2 (S) | 0.5 (I) | <0.12 (S) | <0.12 (S) | 4 (S) | 0.5 (S) | 8 (S) | 4 (S) | 0.5 (S) |
| NX0755 | 16 (I) | 4/2 (S) | 1 (S) | >64 (R) | <0.06/1.2 (S) | 0.5 (I) | <0.12 (S) | <0.12 (S) | 2 (S) | 0.5 (S) | 8 (S) | 2 (S) | 0.5 (S) |
| NX0756 | 16 (I) | 4/2 (S) | 0.5 (S) | >64 (R) | <0.06/1.2 (S) | 0.5 (I) | <0.12 (S) | <0.12 (S) | 4 (S) | 0.5 (S) | 8 (S) | 2 (S) | 0.5 (S) |
| NX08020 | >64 (R) | 2/1 (S) | 0.5 (S) | >64 (R) | <0.06/1.2 (S) | 0.5 (I) | <0.12 (S) | <0.12 (S) | 2 (S) | <0.25 (S) | 8 (S) | <0.5 (S) | 0.5 (S) |
| NX09001 | 16 (I) | 4/2 (S) | >64 (R) | 64 (R) | <0.06/1.2 (S) | 0.5 (I) | 1 (R) | <0.12 (S) | 2 (S) | 0.5 (S) | 8 (S) | 4 (S) | 0.5 (S) |
| NX09002 | 32 (R) | 8/4 (S) | 64 (R) | >64 (R) | 0.06/1.2 (S) | 0.5 (I) | 1 (R) | <0.12 (S) | 2 (S) | <0.25 (S) | 8 (S) | <0.5 (S) | 0.5 (S) |
| NX09003 | 16 (I) | 2/1 (S) | >64 (R) | 64 (R) | <0.06/1.2 (S) | 0.5 (I) | 2 (R) | <0.12 (S) | 4 (S) | 0.5 (S) | 8 (S) | 4 (S) | 0.5 (S) |
| NX09004 | 8 (S) | 2/1 (S) | 8 (I) | 64 (R) | <0.06/1.2 (S) | 0.5 (I) | 0.5 (I) | <0.12 (S) | 2 (S) | 0.5 (S) | 8 (S) | 2 (S) | 0.5 (S) |
| NX09005 | 16 (I) | 2/1 (S) | 16 (R) | 64 (R) | <0.06/1.2 (S) | 0.5 (I) | 2 (R) | <0.12 (S) | 4 (S) | 0.5 (S) | 8 (S) | 4 (S) | 0.5 (S) |
| NX09006 | 32 (R) | 16/8 (I) | >64 (R) | >64 (R) | <0.06/1.2 (S) | 0.5 (I) | 2 (R) | 0.25 (S) | 4 (S) | 1 (S) | 8 (S) | 4 (S) | 0.5 (S) |
| NX09007 | 32 (R) | 16/8 (I) | 1 (S) | 64 (R) | <0.06/1.2 (S) | 1 (I) | <0.12 (S) | 1 (S) | 4 (S) | <0.25 (S) | 2 (S) | 4 (S) | 0.25 (S) |
| NX09008 | 8 (S) | 2/1 (S) | <0.5 (S) | 8 (R) | 0.12/2.4 (S) | 0.5 (I) | <0.12 (S) | <0.12 (S) | 1 (S) | <0.25 (S) | 16 (S) | 2 (S) | 0.5 (S) |
| NX09009 | 16 (I) | 8/4 (S) | 8 (I) | 16 (R) | 0.12/2.4 (S) | 0.5 (I) | 2 (R) | 0.25 (S) | 2 (S) | <0.25 (S) | >128 (R) | 4 (S) | 0.5 (S) |
| NX09010 | 16 (I) | 8/4 (S) | 8 (I) | 16 (R) | 0.25/4.8 (S) | 2 (I) | 2 (R) | 0.25 (S) | 2 (S) | <0.25 (S) | >128 (R) | 4 (S) | 0.5 (S) |
| NX09011 | 16 (I) | 8/4 (S) | 8 (I) | 16 (R) | 0.12/2.4 (S) | 1 (I) | 2 (R) | 0.25 (S) | 2 (S) | <0.25 (S) | >128 (R) | 4 (S) | 0.5 (S) |
| NX09013 | 32 (R) | 8/4 (S) | 1 (S) | 64 (R) | 0.25/4.8 (S) | 0.5 (I) | <0.12 (S) | 0.5 (S) | 4 (S) | <0.25 (S) | 4 (S) | 4 (S) | 0.5 (S) |
| NX09014 | 32 (R) | 16/8 (I) | 1 (S) | 64 (R) | 0.12/2.4 (S) | 1 (I) | <0.12 (S) | 0.5 (S) | 4 (S) | 0.5 (S) | 8 (S) | 8 (S) | 0.5 (S) |
| NX09017 | 32 (R) | 16/8 (I) | 1 (S) | 64 (R) | <0.06/1.2 (S) | 1 (I) | <0.12 (S) | 0.5 (S) | 4 (S) | <0.25 (S) | 8 (S) | 8 (S) | 0.25 (S) |
| NX09018 | 16 (I) | 8/4 (S) | <0.5 (S) | 32 (R) | <0.06/1.2 (S) | 0.5 (I) | <0.12 (S) | 0.25 (S) | 2 (S) | <0.25 (S) | 2 (S) | 4 (S) | 0.5 (S) |
| NX09028 | 8 (S) | 8/4 (S) | 2 (S) | 64 (R) | 0.12/2.4 (S) | 1 (I) | <0.12 (S) | 0.5 (S) | 2 (S) | 1 (S) | 4 (S) | 4 (S) | 0.5 (S) |
| NX09029 | 4 (S) | 4/2 (S) | 1 (S) | 32 (R) | 0.12/2.4 (S) | 1 (I) | <0.12 (S) | <0.12 (S) | 0.5 (S) | <0.25 (S) | 8 (S) | 2 (S) | 0.5 (S) |
| NX09030 | 32 (R) | 2/1 (S) | 16 (R) | 64 (R) | <0.06/1.2 (S) | 0.5 (I) | 2 (R) | <0.12 (S) | 4 (S) | 0.5 (S) | 8 (S) | 4 (S) | 0.5 (S) |
| NX09036 | 32 (R) | 16/8 (I) | 1 (S) | 64 (R) | 0.12/2.4 (S) | 0.5 (I) | <0.12 (S) | 1 (S) | 2 (S) | <0.25 (S) | 4 (S) | 4 (S) | 0.5 (S) |
| NX09037 | 16 (I) | 8/4 (S) | 1 (S) | 32 (R) | 0.12/2.4 (S) | 0.5 (I) | <0.12 (S) | 0.5 (S) | 2 (S) | <0.25 (S) | 8 (S) | 4 (S) | 0.5 (S) |
| **Isolate** | **AMP** | **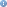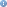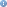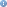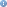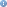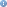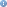SAM** | **TET** | **CZO** | **CXM** | **CAZ** | **SXT** | **POL** | **CIP** | **GEN** | **STR** | **CHL** | **IPM** |
| NX09038 | 32 (R) | 8/4 (S) | 1 (S) | 64 (R) | 0.12/2.4 (S) | 0.5 (I) | <0.12 (S) | 1 (S) | 4 (S) | 0.5 (S) | 4 (S) | 4 (S) | 0.5 (S) |
| NX09039 | 32 (R) | 8/4 (S) | 1 (S) | 32 (R) | 0.12/2.4 (S) | 0.5 (I) | <0.12 (S) | 0.5 (S) | 2 (S) | <0.25 (S) | 4 (S) | 8 (S) | 0.5 (S) |
| NX09040 | 32 (R) | 16/8 (I) | 1 (S) | 64 (R) | 0.12/2.4 (S) | 0.5 (I) | <0.12 (S) | 0.5 (S) | 4 (S) | 0.5 (S) | 4 (S) | 8 (S) | 0.5 (S) |
| NX09041 | 64 (R) | 32/16 (R) | 1 (S) | >64 (R) | 0.12/2.4 (S) | 1 (I) | <0.12 (S) | 1 (S) | 8 (S) | 0.5 (S) | 8 (S) | 8 (S) | 0.5 (S) |
| NX09042 | 32 (R) | 8/4 (S) | 1 (S) | 64 (R) | 0.25/4.8 (S) | 0.5 (I) | <0.12 (S) | 0.25 (S) | 4 (S) | 1 (S) | 4 (S) | 4 (S) | 0.5 (S) |
| NX09043 | 16 (I) | 16/8 (I) | 1 (S) | 32 (R) | 0.12/2.4 (S) | 0.5 (I) | <0.12 (S) | 0.5 (S) | 2 (S) | 0.5 (S) | 8 (S) | 4 (S) | 0.5 (S) |
| NX09044 | 32 (R) | 1/0.5 (S) | 1 (S) | >64 (R) | 0.12/2.4 (S) | 0.5 (I) | <0.12 (S) | 0.5 (S) | 4 (S) | 0.5 (S) | 4 (S) | 4 (S) | 0.5 (S) |
| NX09045 | 32 (R) | 16/8 (I) | 1 (S) | 64 (R) | 0.12/2.4 (S) | 0.5 (I) | <0.12 (S) | 0.5 (S) | 2 (S) | <0.25 (S) | 4 (S) | 4 (S) | 0.5 (S) |
| NX09047 | 16 (I) | 8/4 (S) | <0.5 (S) | 32 (R) | 0.12/2.4 (S) | 0.5 (I) | <0.12 (S) | 0.25 (S) | 2 (S) | <0.25 (S) | 4 (S) | 4 (S) | 0.5 (S) |
| NX09048 | 32 (R) | 16/8 (I) | 1 (S) | 64 (R) | 0.12/2.4 (S) | 0.5 (I) | <0.12 (S) | 0.5 (S) | 2 (S) | <0.25 (S) | 4 (S) | 4 (S) | 0.5 (S) |
| NX09057 | 16 (I) | 8/4 (S) | 1 (S) | 32 (R) | <0.06/1.2 (S) | 0.5 (I) | <0.12 (S) | 0.25 (S) | 2 (S) | <0.25 (S) | 4 (S) | 4 (S) | 0.5 (S) |
| NX09058 | 32 (R) | 16/8 (I) | 1 (S) | 32 (R) | 0.12/2.4 (S) | 0.5 (I) | <0.12 (S) | 0.5 (S) | 4 (S) | <0.25 (S) | 4 (S) | 4 (S) | 0.5 (S) |
| NX09059 | 32 (R) | 16/8 (I) | 1 (S) | 64 (R) | 0.12/2.4 (S) | 0.5 (I) | <0.12 (S) | 1 (S) | 4 (S) | <0.25 (S) | 4 (S) | 4 (S) | 0.5 (S) |
| NX09060 | 32 (R) | 16/8 (I) | 1 (S) | 64 (R) | 0.12/2.4 (S) | 1 (I) | <0.12 (S) | 1 (S) | 4 (S) | 0.5 (S) | 8 (S) | 8 (S) | 0.5 (S) |
| NX09061 | 32 (R) | 16/8 (I) | 1 (S) | 64 (R) | <0.06/1.2 (S) | 0.5 (I) | <0.12 (S) | 0.5 (S) | 4 (S) | 0.5 (S) | 4 (S) | 4 (S) | 0.5 (S) |
| NX09062 | 32 (R) | 16/8 (I) | 1 (S) | >64 (R) | 0.12/2.4 (S) | 0.5 (I) | <0.12 (S) | 0.5 (S) | 4 (S) | <0.25 (S) | 4 (S) | 4 (S) | 0.5 (S) |
| NX10041 | 8 (S) | 8/4 (S) | 1 (S) | 32 (R) | <0.06/1.2 (S) | 0.5 (I) | <0.12 (S) | 0.25 (S) | 1 (S) | <0.25 (S) | 4 (S) | 4 (S) | 0.5 (S) |
| NX10043 | 32 (R) | 16/8 (I) | 1 (S) | >64 (R) | 0.12/2.4 (S) | 0.5 (I) | 2 (R) | 0.25 (S) | 8 (S) | 1 (S) | 8 (S) | 4 (S) | 0.5 (S) |
| NX11036 | 32 (R) | 16/8 (I) | 1 (S) | 64 (R) | 0.12/2.4 (S) | 0.5 (I) | <0.12 (S) | 0.5 (S) | 8 (S) | 0.5 (S) | 4 (S) | 4 (S) | 0.5 (S) |
| NX11058 | 32 (R) | 16/8 (I) | 1 (S) | >64 (R) | 0.12/2.4 (S) | 0.5 (I) | <0.12 (S) | 0.25 (S) | 8 (S) | 1 (S) | 8 (S) | 4 (S) | 0.5 (S) |
| NX11070 | 16 (I) | 8/4 (S) | 1 (S) | >64 (R) | <0.06/1.2 (S) | 0.5 (I) | 0.5 (I) | <0.12 (S) | 4 (S) | 0.5 (S) | 8 (S) | 4 (S) | 0.5 (S) |
| NX11072 | 16 (I) | 8/4 (S) | 1 (S) | >64 (R) | 0.12/2.4 (S) | 0.5 (I) | 1 (R) | 0.25 (S) | 4 (S) | 1 (S) | 8 (S) | 4 (S) | 0.5 (S) |
| NX11073 | 32 (R) | 16/8 (I) | 2 (S) | >64 (R) | 0.12/2.4 (S) | 0.5 (I) | 0.5 (I) | 0.25 (S) | 4 (S) | 1 (S) | 8 (S) | 4 (S) | 0.5 (S) |
| NX11074 | 32 (R) | 16/8 (I) | 1 (S) | >64 (R) | 0.12/2.4 (S) | 0.5 (I) | 1 (R) | 0.25 (S) | 8 (S) | 1 (S) | 8 (S) | 4 (S) | 0.5 (S) |
| NX11075 | 32 (R) | 16/8 (I) | 2 (S) | >64 (R) | 0.12/2.4 (S) | 0.5 (I) | 2 (R) | 0.25 (S) | 8 (S) | 1 (S) | 4 (S) | 4 (S) | 0.5 (S) |
| NX11076 | 32 (R) | 16/8 (I) | 2 (S) | >64 (R) | 0.12/2.4 (S) | 0.5 (I) | 2 (R) | 0.5 (S) | 8 (S) | 1 (S) | 8 (S) | 8 (S) | 0.5 (S) |
| NX11077 | 32 (R) | 8/4 (S) | 1 (S) | >64 (R) | <0.06/1.2 (S) | 0.5 (I) | 0.5 (I) | 0.25 (S) | 4 (S) | 0.5 (S) | 8 (S) | 4 (S) | 0.5 (S) |
| NX11078 | 32 (R) | 32/16 (R) | 1 (S) | >64 (R) | 0.12/2.4 (S) | 0.5 (I) | 2 (R) | 0.25 (S) | 8 (S) | 1 (S) | 8 (S) | 4 (S) | 0.5 (S) |
| NX11079 | 32 (R) | 16/8 (I) | 1 (S) | >64 (R) | <0.06/1.2 (S) | 0.5 (I) | <0.12 (S) | 0.25 (S) | 8 (S) | 1 (S) | 8 (S) | 4 (S) | 0.5 (S) |
| NX11080 | 32 (R) | 16/8 (I) | 1 (S) | >64 (R) | <0.06/1.2 (S) | 0.5 (I) | 2 (R) | 0.25 (S) | 4 (S) | 0.5 (S) | 8 (S) | 4 (S) | 0.5 (S) |
| NX11081 | 16 (I) | 8/4 (S) | 1 (S) | >64 (R) | 0.12/2.4 (S) | 0.5 (I) | 0.5 (I) | <0.12 (S) | 4 (S) | 0.5 (S) | 8 (S) | 4 (S) | 0.5 (S) |
| NX11082 | 32 (R) | 16/8 (I) | 4 (S) | >64 (R) | 0.12/2.4 (S) | 0.5 (I) | 2 (R) | 0.25 (S) | 8 (S) | 1 (S) | 16 (S) | 8 (S) | 0.5 (S) |
| NX11083 | 32 (R) | 8/4 (S) | 2 (S) | >64 (R) | 0.12/2.4 (S) | 0.5 (I) | 0.5 (I) | 0.25 (S) | 8 (S) | 1 (S) | 8 (S) | 8 (S) | 0.5 (S) |
| NX11084 | 32 (R) | 8/4 (S) | 2 (S) | 64 (R) | 0.12/2.4 (S) | 0.5 (I) | 0.5 (I) | 0.25 (S) | 4 (S) | 1 (S) | 16 (S) | 8 (S) | 0.5 (S) |
| NX11085 | 32 (R) | 8/4 (S) | 1 (S) | >64 (R) | <0.06/1.2 (S) | 0.5 (I) | 2 (R) | <0.12 (S) | 4 (S) | 0.5 (S) | 8 (S) | 4 (S) | 0.5 (S) |
| NX11086 | 16 (I) | 8/4 (S) | 2 (S) | >64 (R) | <0.06/1.2 (S) | 0.5 (I) | 0.5 (I) | <0.12 (S) | 4 (S) | 1 (S) | 8 (S) | 4 (S) | 0.5 (S) |
| NX11087 | 16 (I) | 4/2 (S) | <0.5 (S) | >64 (R) | <0.06/1.2 (S) | 0.5 (I) | <0.12 (S) | <0.12 (S) | 4 (S) | 0.5 (S) | 8 (S) | 4 (S) | 0.5 (S) |
| NX11088 | 32 (R) | 8/4 (S) | >64(R) | >64 (R) | 2/38 (S) | 1 (I) | <0.12 (S) | <0.12 (S) | 4 (S) | 0.5 (S) | >128 (R) | 4 (S) | 0.5 (S) |
| NX11089 | 32 (R) | 8/4 (S) | 2 (S) | >64 (R) | <0.06/1.2 (S) | 1 (I) | 2 (R) | 0.25 (S) | 4 (S) | 1 (S) | 8 (S) | 4 (S) | 0.5 (S) |
| NX11090 | 32 (R) | 8/4 (S) | 2 (S) | >64 (R) | <0.06/1.2 (S) | 0.5 (I) | 2 (R) | 0.25 (S) | 4 (S) | 1 (S) | 8 (S) | 4 (S) | 0.5 (S) |
| **Isolate** | **AMP** | **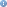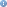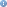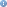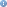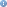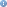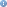SAM** | **TET** | **CZO** | **CXM** | **CAZ** | **SXT** | **POL** | **CIP** | **GEN** | **STR** | **CHL** | **IPM** |
| NX11091 | 32 (R) | 16/8 (I) | 2 (S) | >64 (R) | 0.12/2.4 (S) | 0.5 (I) | 0.5 (I) | 0.25 (S) | 8 (S) | 1 (S) | 8 (S) | 8 (S) | 0.5 (S) |
| NX11092 | 16 (I) | 8/4 (S) | <0.5 (S) | >64 (R) | <0.06/1.2 (S) | 0.25 (I) | <0.12 (S) | <0.12 (S) | 4 (S) | <0.25 (S) | 8 (S) | 2 (S) | 0.5 (S) |
| NX11093 | 32 (R) | 16/8 (I) | 4 (S) | >64 (R) | 0.12/2.4 (S) | 0.5 (I) | 2 (R) | 0.5 (S) | 8 (S) | 1 (S) | 8 (S) | 8 (S) | 0.5 (S) |
| NX11094 | 32 (R) | 8/4 (S) | 1 (S) | >64 (R) | <0.06/1.2 (S) | 0.25 (I) | <0.12 (S) | 0.25 (S) | 4 (S) | 0.5 (S) | 8 (S) | 4 (S) | 0.25 (S) |
| NX12009 | 32 (R) | 16/8 (I) | 1 (S) | 32 (R) | <0.06/1.2 (S) | 0.5 (I) | <0.12 (S) | 1 (S) | 4 (S) | <0.25 (S) | 8 (S) | 4 (S) | 0.5 (S) |
| NX12010 | 16 (I) | 8/4 (S) | 1 (S) | 32 (R) | 0.12/2.4 (S) | 0.25 (I) | <0.12 (S) | 0.5 (S) | 2 (S) | <0.25 (S) | 4 (S) | 4 (S) | 0.5 (S) |
| NX12011 | 16 (I) | 8/4 (S) | 1 (S) | 32 (R) | 0.12/2.4 (S) | 0.5 (I) | <0.12 (S) | 0.5 (S) | 4 (S) | <0.25 (S) | 4 (S) | 4 (S) | 0.5 (S) |
| NX12012 | 8 (S) | 2/1 (S) | <0.5 (S) | 4 (I) | <0.06/1.2 (S) | 0.5 (I) | <0.12 (S) | <0.12 (S) | 1 (S) | <0.25 (S) | 4 (S) | 4 (S) | 0.5 (S) |
| NX12013 | 8 (S) | 4/2 (S) | <0.5 (S) | 8 (R) | <0.06/1.2 (S) | 0.5 (I) | <0.12 (S) | 0.25 (S) | 1 (S) | <0.25 (S) | 4 (S) | 4 (S) | 0.5 (S) |
| NX12014 | 16 (I) | 8/4 (S) | 1 (S) | 32 (R) | 0.12/2.4 (S) | 1 (I) | <0.12 (S) | 0.25 (S) | 4 (S) | <0.25 (S) | 4 (S) | 4 (S) | 0.5 (S) |
| NX12015 | 16 (I) | 4/2 (S) | <0.5 (S) | 32 (R) | 0.12/2.4 (S) | 0.5 (I) | <0.12 (S) | 0.25 (S) | 4 (S) | <0.25 (S) | 2 (S) | 4 (S) | 0.5 (S) |
| NX12016 | 16 (I) | 8/4 (S) | <0.5 (S) | 16 (R) | 0.12/2.4 (S) | 0.5 (I) | 0.25 (S) | 0.25 (S) | 2 (S) | <0.25 (S) | 4 (S) | 4 (S) | 0.5 (S) |
| NX12017 | 16 (I) | 8/4 (S) | <0.5 (S) | 32 (R) | <0.06/1.2 (S) | 0.25 (I) | <0.12 (S) | 0.25 (S) | 2 (S) | <0.25 (S) | 8 (S) | 4 (S) | 0.5 (S) |
| NX12018 | 16 (I) | 4/2 (S) | 1 (S) | 8 (R) | <0.06/1.2 (S) | 0.5 (I) | 0.25 (S) | <0.12 (S) | 1 (S) | <0.25 (S) | 2 (S) | 4 (S) | 0.5 (S) |
| NX12019 | 16 (I) | 8/4 (S) | 1 (S) | 16 (R) | 0.12/2.4 (S) | 0.5 (I) | 0.5 (I) | 0.25 (S) | 2 (S) | <0.25 (S) | 4 (S) | 4 (S) | 0.5 (S) |
| NX12020 | 16 (I) | 4/2 (S) | <0.5 (S) | 32 (R) | 0.12/2.4 (S) | 0.5 (I) | <0.12 (S) | 0.25 (S) | 2 (S) | <0.25 (S) | 8 (S) | 4 (S) | 0.5 (S) |
| NX12023 | 32 (R) | 8/4 (S) | 1 (S) | 32 (R) | <0.06/1.2 (S) | 0.5 (I) | <0.12 (S) | 0.5 (S) | 4 (S) | <0.25 (S) | 4 (S) | 4 (S) | 0.5 (S) |
| NX12024 | 16 (I) | 4/2 (S) | 1 (S) | 16 (R) | 0.12/2.4 (S) | 0.5 (I) | <0.12 (S) | 0.25 (S) | 2 (S) | <0.25 (S) | 4 (S) | 4 (S) | 0.5 (S) |
| NX12025 | 32 (R) | 8/4 (S) | 1 (S) | 32 (R) | 0.12/2.4 (S) | 0.5 (I) | <0.12 (S) | 0.25 (S) | 4 (S) | <0.25 (S) | 4 (S) | 4 (S) | 0.5 (S) |
| NX12036 | 16 (I) | 8/4 (S) | <0.5 (S) | 32 (R) | 0.12/2.4 (S) | 0.25 (I) | <0.12 (S) | 0.25 (S) | 2 (S) | <0.25 (S) | 4 (S) | 4 (S) | 0.5 (S) |
| NX12056 | 16 (I) | 8/4 (S) | <0.5 (S) | >64 (R) | 0.12/2.4 (S) | 0.5 (I) | 2 (R) | <0.12 (S) | 4 (S) | <0.25 (S) | 8 (S) | 4 (S) | 0.5 (S) |
| NX12057 | 16 (I) | 8/4 (S) | 1 (S) | 64 (R) | <0.06/1.2 (S) | 0.5 (I) | 0.5 (I) | <0.12 (S) | 4 (S) | 1 (S) | 8 (S) | 4 (S) | 0.5 (S) |
| NX12058 | 16 (I) | 8/4 (S) | 64 (R) | >64 (R) | 2/38 (S) | 0.5 (I) | <0.12 (S) | <0.12 (S) | 4 (S) | 0.5 (S) | >128 (R) | 4 (S) | 0.5 (S) |
| NX12059 | 16 (I) | 8/4 (S) | 1 (S) | >64 (R) | <0.06/1.2 (S) | 0.5 (I) | 0.5 (I) | <0.12 (S) | 4 (S) | 1 (S) | 8 (S) | 4 (S) | 0.5 (S) |
| NX12060 | 16 (I) | 8/4 (S) | >64 (R) | >64 (R) | 2/38 (S) | 0.5 (I) | <0.12 (S) | <0.12 (S) | 4 (S) | 0.5 (S) | >128 (R) | 4 (S) | 0.5 (S) |
| NX12061 | 16 (I) | 8/4 (S) | >64 (R) | >64 (R) | 2/38 (S) | 0.5 (I) | <0.12 (S) | <0.12 (S) | 4 (S) | 0.5 (S) | >128 (R) | 4 (S) | 0.5 (S) |
| NX12062 | 16 (I) | 8/4 (S) | >64 (R) | >64 (R) | 2/38 (S) | 0.5 (I) | <0.12 (S) | <0.12 (S) | 2 (S) | 0.5 (S) | >128 (R) | 2 (S) | 0.25 (S) |
| NX12063 | 16 (I) | 4/2 (S) | <0.5 (S) | >64 (R) | <0.06/1.2 (S) | 0.25 (I) | <0.12 (S) | <0.12 (S) | 2 (S) | 0.5 (S) | 4 (S) | 2 (S) | 0.5 (S) |
| NX12064 | 32 (R) | 8/4 (S) | 1 (S) | 32 (R) | 0.12/2.4 (S) | 0.5 (I) | <0.12 (S) | 0.5 (S) | 4 (S) | <0.25 (S) | 4 (S) | 4 (S) | 0.5 (S) |
| NX12065 | 16 (I) | 4/2 (S) | 1 (S) | 8 (R) | 0.12/2.4 (S) | 0.5 (I) | 0.5 (I) | 0.25 (S) | 2 (S) | <0.25 (S) | 4 (S) | 4 (S) | 0.5 (S) |
| NX13049 | 16 (I) | 8/4 (S) | 64 (R) | 32 (R) | 1/19 (S) | 1 (I) | 0.5 (I) | 0.25 (S) | 2 (S) | 0.5 (S) | >128 (R) | 32 (R) | 0.25 (S) |
| NX14046 | 32 (R) | 8/4 (S) | 1 (S) | 32 (R) | <0.06/1.2 (S) | 1 (I) | <0.12 (S) | 0.5 (S) | 4 (S) | <0.25 (S) | 4 (S) | 4 (S) | 0.5 (S) |
| NX14047 | 32 (R) | 16/8 (I) | 1 (S) | 32 (R) | <0.06/1.2 (S) | 0.5 (I) | <0.12 (S) | 0.5 (S) | 4 (S) | <0.25 (S) | 4 (S) | 4 (S) | 0.5 (S) |
| NX14050 | 32 (R) | 16/8 (I) | 1 (S) | 64 (R) | <0.06/1.2 (S) | 1 (I) | <0.12 (S) | 1 (S) | 8 (S) | <0.25 (S) | 4 (S) | 4 (S) | 0.5 (S) |
| NX14056 | 32 (R) | 16/8 (I) | 1 (S) | >64 (R) | <0.06/1.2 (S) | 0.5 (I) | <0.12 (S) | 1 (S) | 8 (S) | <0.25 (S) | 4 (S) | 4 (S) | 0.5 (S) |
| NX14109 | 16 (I) | 2/1 (S) | <0.5 (S) | 16 (R) | <0.06/1.2 (S) | 0.5 (I) | <0.12 (S) | <0.12 (S) | 1 (S) | <0.25 (S) | 4 (S) | 1 (S) | 0.5 (S) |
| NX14111 | 16 (I) | 2/1 (S) | <0.5 (S) | 32 (R) | <0.06/1.2 (S) | 0.5 (I) | <0.12 (S) | <0.12 (S) | 2 (S) | <0.25 (S) | 4 (S) | 1 (S) | 0.5 (S) |
| NX14112 | 16 (I) | 4/2 (S) | <0.5 (S) | 16 (R) | <0.06/1.2 (S) | 0.5 (I) | <0.12 (S) | <0.12 (S) | 1 (S) | <0.25 (S) | 4 (S) | <0.5 (S) | 0.5 (S) |
| NX14113 | 16 (I) | 8/4 (S) | 1 (S) | 32 (R) | <0.06/1.2 (S) | 0.5 (I) | <0.12 (S) | 1 (S) | 4 (S) | <0.25(S) | 4 (S) | 4 (S) | 0.5 (S) |
| NX14114 | 64 (R) | 16/8 (I) | <0.5 (S) | 32 (R) | <0.06/1.2 (S) | 0.5 (I) | <0.12 (S) | 2 (S) | 16 (I) | <0.25 (S) | 4 (S) | 4 (S) | 0.5 (S) |
| **Isolate** | **AMP** | **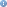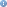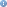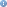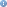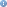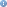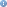SAM** | **TET** | **CZO** | **CXM** | **CAZ** | **SXT** | **POL** | **CIP** | **GEN** | **STR** | **CHL** | **IPM** |
| NX14115 | 16 (I) | 2/1 (S) | 1 (S) | 32 (R) | <0.06/1.2 (S) | 0.5 (I) | <0.12 (S) | <0.12 (S) | 2 (S) | <0.5 (S) | 4 (S) | 2 (S) | 0.25 (S) |
| NX14116 | 16 (I) | 2/1 (S) | <0.5 (S) | 64 (R) | <0.06/1.2 (S) | 1 (I) | <0.12 (S) | <0.12 (S) | 2 (S) | <0.25 (S) | 2 (S) | 1 (S) | 1 (S) |
| NX14117 | 16 (I) | 2/1 (S) | <0.5 (S) | 32 (R) | <0.06/1.2 (S) | 0.5 (I) | <0.12 (S) | <0.12 (S) | 1 (S) | 0.5 (S) | 2 (S) | 1 (S) | 0.5 (S) |
| NX14118 | 16 (I) | 2/1 (S) | <0.5 (S) | 16 (R) | <0.06/1.2 (S) | 0.5 (I) | <0.12 (S) | <0.12 (S) | 1 (S) | <0.25 (S) | 4 (S) | 1 (S) | 0.5 (S) |
| NX14121 | 32 (R) | 32/16 (R) | 1 (S) | 64 (R) | <0.06/1.2 (S) | 0.5 (I) | <0.12 (S) | 1 (S) | 16 (I) | 0.5 (S) | 4 (S) | 4 (S) | 0.5 (S) |
| NX15095 | 16 (I) | 8/4 (S) | 1 (S) | 32 (R) | <0.06/1.2 (S) | 0.5 (I) | <0.12 (S) | 0.5 (S) | 1 (S) | <0.25 (S) | 4 (S) | 4 (S) | 0.5 (S) |
| NX15096 | 16 (I) | 4/2 (S) | 1 (S) | 16 (R) | 0.12/2.4 (S) | 0.5 (I) | <0.12 (S) | 0.25 (S) | 2 (S) | <0.25 (S) | 2 (S) | 4 (S) | 0.5 (S) |
| NX15097 | 16 (I) | 4/2 (S) | 1 (S) | 16 (R) | 0.12/2.4 (S) | 0.5 (I) | <0.12 (S) | 0.25 (S) | 2 (S) | <0.25 (S) | 4 (S) | 4 (S) | 0.5 (S) |
| NX15098 | 16 (I) | 4/2 (S) | 1 (S) | 16 (R) | 0.12/2.4 (S) | 0.5 (I) | <0.12 (S) | 0.25 (S) | 2 (S) | <0.25 (S) | 2 (S) | 4 (S) | 0.5 (S) |
| NX15099 | 32 (R) | 16/8 (I) | 1 (S) | 32 (R) | <0.06/1.2 (S) | 0.5 (I) | <0.12 (S) | 0.5 (S) | 4 (S) | <0.25 (S) | 4 (S) | 4 (S) | 0.5 (S) |
| NX15100 | 16 (I) | 4/2 (S) | 1 (S) | 16 (R) | 0.12/2.4 (S) | 0.5 (I) | <0.12 (S) | 0.25 (S) | 2 (S) | <0.25 (S) | 2 (S) | 4 (S) | 0.5 (S) |
| NX15106 | 16 (I) | 8/4 (S) | 1 (S) | 16 (R) | <0.06/1.2 (S) | 0.5 (I) | <0.12 (S) | 0.25 (S) | 2 (S) | <0.25 (S) | 4 (S) | 4 (S) | 0.5 (S) |
| NX15108 | 16 (I) | 4/2 (S) | <0.5 (S) | 16 (R) | <0.06/1.2 (S) | 0.5 (I) | <0.12 (S) | 0.25 (S) | 2 (S) | <0.25 (S) | 4 (S) | 4 (S) | 0.5 (S) |
| NX15132 | 16 (I) | 8/4 (S) | <0.5 (S) | 32 (R) | 0.12/2.4 (S) | 0.5 (I) | <0.12 (S) | 0.25 (S) | 2 (S) | <0.25 (S) | 2 (S) | 4 (S) | 0.5 (S) |
| NX16034 | 16 (I) | 8/4 (S) | 1 (S) | 16 (R) | <0.06/1.2 (S) | 0.5 (I) | <0.12 (S) | 0.5 (S) | 2 (S) | <0.25 (S) | 4 (S) | 4 (S) | 0.5 (S) |
| NX16044 | 64 (R) | 16/8 (I) | 1 (S) | 32 (R) | <0.06/1.2 (S) | 0.5 (I) | <0.12 (S) | 2 (S) | 8 (S) | <0.25 (S) | 2 (S) | 4 (S) | 0.5 (S) |
| NX16081 | 32 (R) | 16/8 (I) | 1 (S) | >64 (R) | 2/38 (S) | 0.5 (I) | 1 (R) | 0.25 (S) | 4 (S) | <0.5 (S) | 8 (S) | 4 (S) | 0.5 (S) |
| NX16082 | 32 (R) | 16/8 (I) | 1 (S) | >64 (R) | 2/38 (S) | 0.5 (I) | 1 (R) | 0.5 (S) | 4 (S) | <0.5 (S) | 8 (S) | 4 (S) | 0.5 (S) |
| NX16083 | 16 (I) | 16/8 (I) | 1 (S) | >64 (R) | 2/38 (S) | 0.5 (I) | 1 (R) | 0.25 (S) | 4 (S) | 1 (S) | 8 (S) | 4 (S) | 0.5 (S) |
| NX16084 | 32 (R) | 16/8 (I) | 2 (S) | >64 (R) | 2/38 (S) | 1 (I) | 2 (R) | 0.25 (S) | 4 (S) | 0.5 (S) | 8 (S) | 4 (S) | 0.5 (S) |
| NX16106 | 16 (I) | 8/4 (S) | 1 (S) | 32 (R) | 0.12/2.4 (S) | 0.5 (I) | 0.5 (I) | 0.25 (S) | 2 (S) | <0.25 (S) | 2 (S) | 4 (S) | 0.5 (S) |
| NX17027 | 32 (R) | 8/4 (S) | 1 (S) | 32 (R) | 0.12/2.4 (S) | 1 (I) | <0.12 (S) | 0.25 (S) | 2 (S) | <0.25 (S) | 2 (S) | 4 (S) | 0.5 (S) |
| NX17061 | 16 (I) | 8/4 (S) | 1 (S) | 16 (R) | <0.06/1.2 (S) | 1 (I) | <0.12 (S) | 0.25 (S) | 2 (S) | <0.25 (S) | 4 (S) | 4 (S) | 0.5 (S) |
| NX17062 | 32 (R) | 8/4 (S) | 1 (S) | 32 (R) | 0.12/2.4 (S) | 1 (I) | <0.12 (S) | 0.5 (S) | 2 (S) | 0.5 (S) | 4 (S) | 4 (S) | 0.5 (S) |
| NX17063 | 16 (I) | 8/4 (S) | 1 (S) | 16 (R) | 0.12/2.4 (S) | 1 (I) | <0.12 (S) | 0.25 (S) | 4 (S) | <0.25 (S) | 2 (S) | 4 (S) | 0.5 (S) |
| NX17064 | 16 (I) | 8/4 (S) | 1 (S) | 16 (R) | 0.12/2.4 (S) | 2 (I) | <0.12 (S) | 0.25 (S) | 2 (S) | <0.25 (S) | 4 (S) | 4 (S) | 0.5 (S) |
| NX17067 | 16 (I) | 8/4 (S) | 1 (S) | 16 (R) | 0.12/2.4 (S) | 1 (I) | <0.12 (S) | 0.25 (S) | 2 (S) | <0.25 (S) | 4 (S) | 4 (S) | 0.5 (S) |
| NX17071 | 32 (R) | 8/4 (S) | 1 (S) | 64 (R) | 0.12/2.4 (S) | 1 (I) | <0.12 (S) | 0.5 (S) | 4 (S) | 0.5 (S) | 4 (S) | 8 (S) | 0.25 (S) |
| NX17074 | 16 (I) | 8/4 (S) | 1 (S) | 32 (R) | <0.06/1.2 (S) | 1 (I) | <0.12 (S) | 0.5 (S) | 2 (S) | <0.25 (S) | 4 (S) | 4 (S) | 0.5 (S) |
| NX18077 | 16 (I) | 8/4 (S) | 1 (S) | 32 (R) | 0.12/2.4 (S) | 0.5 (I) | 0.5 (I) | 0.25 (S) | 2 (S) | 0.5 (S) | 4 (S) | 8 (S) | 0.5 (S) |
| NX18080 | 32 (R) | 8/4 (S) | 1 (S) | 32 (R) | 0.12/2.4 (S) | 0.5 (I) | 0.25 (S) | 0.5 (S) | 4 (S) | <0.25 (S) | 2 (S) | 8 (S) | 0.5 (S) |
| NX18083 | 8 (S) | 8/4 (S) | 1 (S) | 8 (R) | <0.06/1.2 (S) | 0.5 (I) | <0.12 (S) | 0.25 (S) | 1 (S) | <0.25 (S) | 4 (S) | 8 (S) | 0.5 (S) |
| NX18084 | 32 (R) | 16/8 (I) | 64 (R) | >64 (R) | 2/38 (S) | 0.5 (I) | 1 (R) | 8 (I) | 16 (I) | 1 (S) | >128 (R) | >64 (R) | 0.5 (S) |
| NX18085 | 32 (R) | 16/8 (I) | >64 (R) | >64 (R) | 4/76 (R) | 0.5 (I) | 2 (R) | 0.25 (S) | 4 (S) | 0.5 (S) | 8 (S) | 4 (S) | 0.5 (S) |
| NX18106 | 8 (S) | 4/2 (S) | <0.5 (S) | 4 (I) | <0.06/1.2 (S) | 0.5 (I) | <0.12 (S) | <0.12 (S) | 1 (S) | <0.25 (S) | 2 (S) | 4 (S) | 0.5 (S) |
| NX18107 | 16 (I) | 8/4 (S) | 1 (S) | 32 (R) | <0.06/1.2 (S) | 0.5 (I) | <0.12 (S) | 0.25 (S) | 2 (S) | <0.25 (S) | 2 (S) | 4 (S) | 0.5 (S) |
| NX18108 | 32 (R) | 16/8 (I) | 64 (R) | >64 (R) | 2/38 (S) | 0.5 (I) | <0.12 (S) | 1 (S) | 8 (S) | 0.5 (S) | >128 (R) | >64 (R) | 0.5 (S) |
| NX18109 | 64 (R) | 16/8 (I) | 64 (R) | >64 (R) | 2/38 (S) | 0.5 (I) | 2 (R) | 8 (I) | 16 (I) | 1 (S) | >128 (R) | >64 (R) | 0.5 (S) |
| NX18110 | 64 (R) | 16/8 (I) | 64 (R) | >64 (R) | 4/76 (R) | 0.5 (I) | 2 (R) | 8 (I) | 16 (I) | >32 (R) | >128 (R) | >64 (R) | 0.5 (S) |
| NX18118 | 32 (R) | 8/4 (S) | >64 (R) | >64 (R) | 4/76 (R) | 0.5 (I) | 2 (R) | 0.5 (S) | 4 (S) | 2 (S) | >128 (R) | >64 (R) | 0.5 (S) |
| **Isolate** | **AMP** | **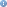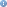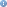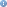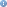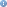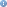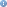SAM** | **TET** | **CZO** | **CXM** | **CAZ** | **SXT** | **POL** | **CIP** | **GEN** | **STR** | **CHL** | **IPM** |
| NX18160 | 32 (R) | 16/8 (I) | 1 (S) | >64 (R) | 4/76 (R) | 0.5 (I) | 2 (R) | <0.12 (S) | 4 (S) | 1 (S) | 8 (S) | 4 (S) | 0.5 (S) |
| NX18161 | 32 (R) | 8/4 (S) | 1 (S) | >64 (R) | 2/38 (S) | 0.5 (I) | 2 (R) | <0.12 (S) | 4 (S) | 1 (S) | 8 (S) | 4 (S) | 0.5 (S) |
| NX18162 | 32 (R) | 8/4 (S) | 1 (S) | >64 (R) | 0.12/2.4 (S) | 0.5 (I) | 2 (R) | <0.12 (S) | 4 (S) | 1 (S) | 8 (S) | 4 (S) | 0.5 (S) |
| NX18163 | 32 (R) | 8/4 (S) | 1 (S) | >64 (R) | 2/38 (S) | 0.5 (I) | 1 (R) | <0.12 (S) | 4 (S) | 1 (S) | 8 (S) | 4 (S) | 0.5 (S) |
| NX18164 | 16 (I) | 8/4 (S) | 1 (S) | >64 (R) | 8/152 (R) | 0.5 (I) | 2 (R) | <0.12 (S) | 4 (S) | 1 (S) | 8 (S) | 4 (S) | 0.5 (S) |
| NX18165 | 32 (R) | 16/8 (I) | 2 (S) | >64 (R) | 4/76 (R) | 0.5 (I) | 2 (R) | 0.5 (S) | 8 (S) | 1 (S) | 8 (S) | 4 (S) | 0.25 (S) |
| NX18166 | 32 (R) | 8/4 (S) | 1 (S) | >64 (R) | 2/38 (S) | 0.5 (I) | 1 (R) | <0.12 (S) | 4 (S) | 1 (S) | 8 (S) | 4 (S) | 0.5 (S) |
| NX18167 | 32 (R) | 16/8 (I) | 1 (S) | >64 (R) | 4/76 (R) | 1 (I) | 2 (R) | <0.12 (S) | 4 (S) | 1 (S) | 8 (S) | 4 (S) | 0.25 (S) |
| NX18168 | 32 (R) | 16/8 (I) | 2 (S) | >64 (R) | 0.12/2.4 (S) | 1 (I) | 2 (R) | 0.25 (S) | 8 (S) | 1 (S) | 8 (S) | 4 (S) | 0.5 (S) |
| NX18169 | 32 (R) | 16/8 (I) | 1 (S) | >64 (R) | 4/76 (R) | 0.5 (I) | 2 (R) | <0.12 (S) | 8 (S) | 1 (S) | 8 (S) | 4 (S) | 0.5 (S) |
| NX18170 | 32 (R) | 16/8 (I) | 1 (S) | >64 (R) | 2/38 (S) | 0.5 (I) | 2 (R) | <0.12 (S) | 4 (S) | 0.5 (S) | 8 (S) | 4 (S) | 0.5 (S) |
| NX18171 | 32 (R) | 16/8 (I) | 2 (S) | >64 (R) | 4/76 (R) | 1 (I) | 2 (R) | 0.25 (S) | 8 (S) | 1 (S) | 8 (S) | 8 (S) | 0.5 (S) |
| NX18172 | 32 (R) | 16/8 (I) | 1 (S) | >64 (R) | 4/76 (R) | 1 (I) | 2 (R) | <0.12 (S) | 4 (S) | 1 (S) | 8 (S) | 4 (S) | 0.5 (S) |
| NX18176 | 32 (R) | 16/8 (I) | 1 (S) | >64 (R) | 4/76 (R) | 0.5 (I) | 2 (R) | 0.25 (S) | 8 (S) | 1 (S) | 8 (S) | 4 (S) | 0.5 (S) |
| NX19005 | 16 (I) | 8/4 (S) | 1 (S) | 32 (R) | 0.25/4.8 (S) | 0.5 (I) | <0.12 (S) | 0.25 (S) | 2 (S) | <0.25 (S) | 4 (S) | 8 (S) | 0.5 (S) |
| NX19006 | 16 (I) | 8/4 (S) | 1 (S) | 16 (R) | <0.06/1.2 (S) | 1 (I) | <0.12 (S) | 0.25 (S) | 2 (S) | <0.25 (S) | 4 (S) | 4 (S) | 0.5 (S) |
| NX19018 | 16 (I) | 8/4 (S) | 8 (I) | 16 (R) | 0.12/2.4 (S) | 0.5 (I) | 2 (R) | 0.25 (S) | 2 (S) | 0.5 (S) | >128 (R) | >64 (R) | 0.25 (S) |
| NX19019 | 16 (I) | 4/2 (S) | <0.5 (S) | >64 (R) | <0.06/1.2 (S) | 0.5 (I) | <0.12 (S) | <0.12 (S) | 2 (S) | 1 (S) | 8 (S) | 2 (S) | 0.5 (S) |
| NX19029 | 16 (I) | 8/4 (S) | 1 (S) | 32 (R) | <0.06/1.2 (S) | 0.5 (I) | <0.12 (S) | 0.25 (S) | 2 (S) | <0.25 (S) | 4 (S) | 4 (S) | 0.5 (S) |
| NX19042 | 32 (R) | 16/8 (I) | 1 (S) | 32 (R) | 0.12/2.4 (S) | 0.5 (I) | <0.12 (S) | 0.5 (S) | 4 (S) | <0.25 (S) | 4 (S) | 4 (S) | 0.5 (S) |
| NX19043 | 32 (R) | 16/8 (I) | 1 (S) | 32 (R) | 0.12/2.4 (S) | 0.5 (I) | <0.12 (S) | 0.5 (S) | 4 (S) | <0.25 (S) | 4 (S) | 4 (S) | 0.5 (S) |
| NX19045 | 16 (I) | 8/4 (S) | 1 (S) | 32 (R) | 0.25/4.8 (S) | 1 (I) | <0.12 (S) | 0.25 (S) | 2 (S) | <0.25 (S) | 2 (S) | 8 (S) | 0.5 (S) |
| NX19048 | 16 (I) | 2/1 (S) | <0.5 (S) | 8 (R) | <0.06/1.2 (S) | 1 (I) | <0.12 (S) | <0.12 (S) | 2 (S) | 0.5 (S) | 4 (S) | 4 (S) | 0.5 (S) |
| NX19053 | 32 (R) | 16/8 (I) | 1 (S) | 32 (R) | <0.06/1.2 (S) | 0.5 (I) | <0.12 (S) | 0.5 (S) | 4 (S) | <0.25 (S) | 4 (S) | 4 (S) | 0.5 (S) |
| NX19054 | 16 (I) | 8/4 (S) | 1 (S) | 32 (R) | <0.06/1.2 (S) | 0.5 (I) | <0.12 (S) | 0.5 (S) | 2 (S) | <0.25 (S) | 2 (S) | 8 (S) | 0.5 (S) |
| NX19106 | 16 (I) | 8/4 (S) | 1 (S) | 32 (R) | 0.12/2.4 (S) | 0.5 (I) | 0.5 (I) | 1 (S) | 2 (S) | <0.25 (S) | 2 (S) | 4 (S) | 0.5 (S) |
| NX19107 | 16 (I) | 8/4 (S) | <0.5 (S) | 32 (R) | 0.12/2.4 (S) | 0.5 (I) | 0.5 (I) | 0.25 (S) | 2 (S) | <0.25 (S) | 4 (S) | 4 (S) | 0.5 (S) |
| NX19109 | 16 (I) | 8/4 (S) | 1 (S) | 32 (R) | <0.06/1.2 (S) | 0.5 (I) | 0.25 (S) | 0.5 (S) | 4 (S) | <0.25 (S) | 2 (S) | 4 (S) | 0.5 (S) |
| NX19111 | 16 (I) | 8/4 (S) | <0.5 (S) | 32 (R) | <0.06/1.2 (S) | 1 (I) | 0.5 (I) | 0.25 (S) | 2 (S) | <0.25 (S) | 4 (S) | 4 (S) | 0.5 (S) |
| NX19112 | 16 (I) | 8/4 (S) | 1 (S) | 16 (R) | <0.06/1.2 (S) | 1 (I) | <0.12 (S) | 0.25 (S) | 2 (S) | <0.25 (S) | 4 (S) | 4 (S) | 0.5 (S) |

AMP, ampicillin; SAM, ampicillin/sulbactam, TET, tetracycline; CZO,cefazolin; CXM, cefuroxime; CAZ, ceftazidime; SXT, trimethoprim/sulfamethoxazole; POL, polymyxin; CIP, ciprofloxacin; GEN, gentamicin; STR, streptomycin; CHL, chloramphenicol; IPM, imipenem; R, resistant; S, susceptible; I, intermediate resistant.

TABLE S6. List of resistance determinants
