# Supplementary figures and images for: Whole-genome sequencing-based prediction and analysis of antimicrobial resistance in Yersinia enterocolitica from Ningxia, China
Source: Front Microbiol. 2022 Jul 22;13:936425. doi: 10.3389/fmicb.2022.936425 (PMC9356307; doi:10.3389/fmicb.2022.936425)

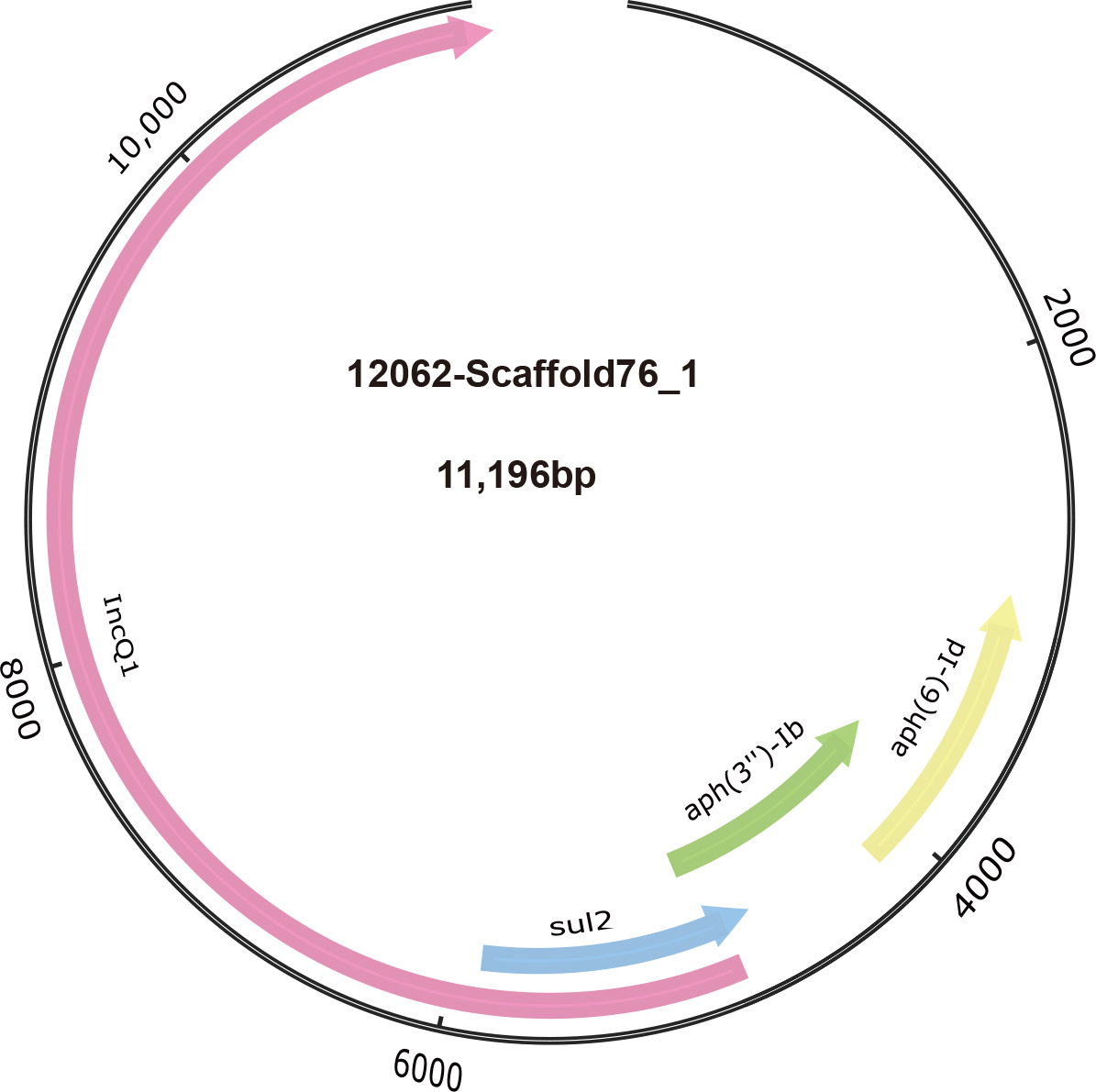

Supplement: Supplementary file 3 [file Image_1.JPEG]

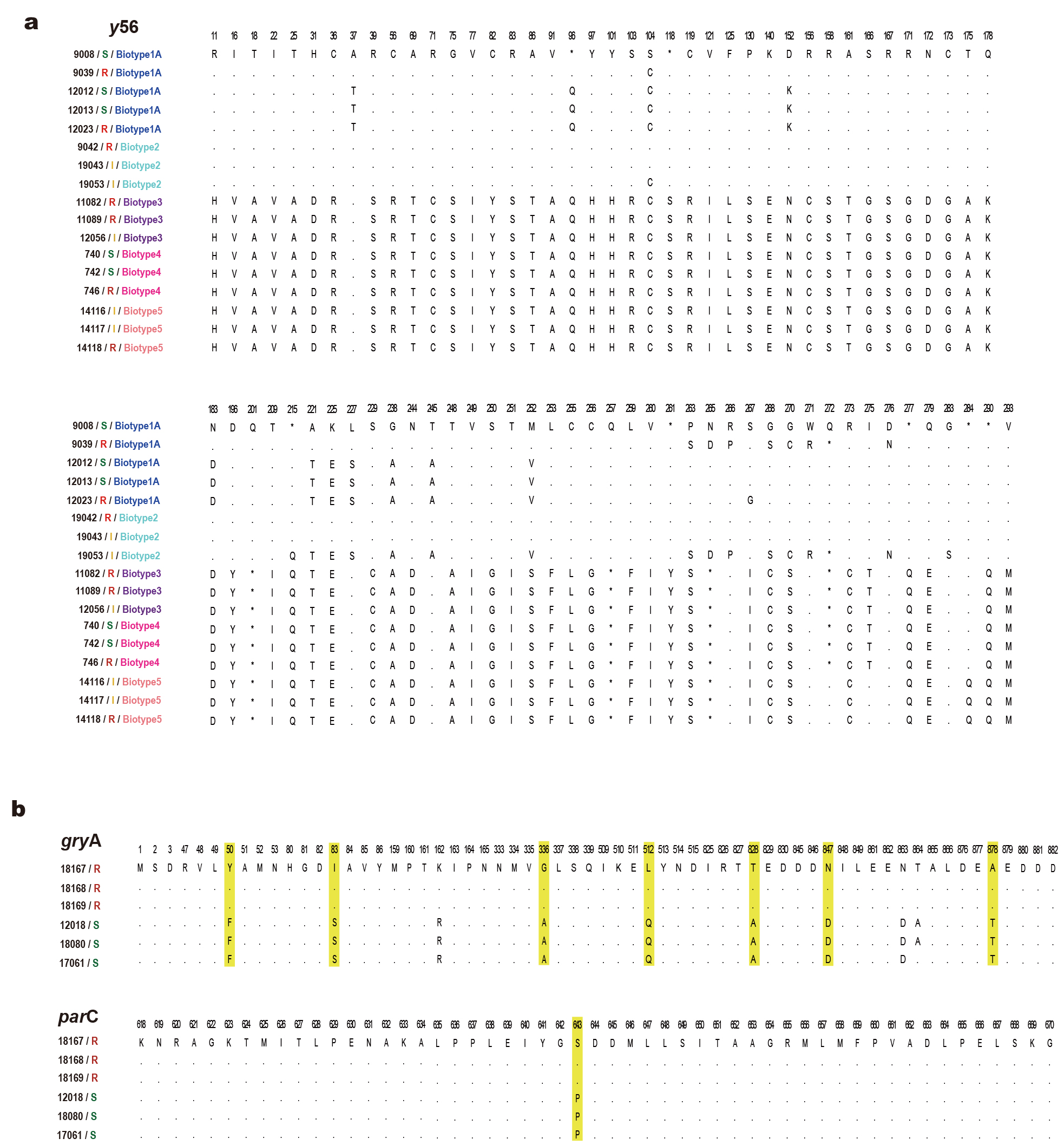

Supplement: Supplementary file 4 [file Image_2.JPEG]
